# Climate‐Driven Variability and Trends in Plant Productivity Over Recent Decades Based on Three Global Products

**DOI:** 10.1029/2020GB006613

**Published:** 2020-12-08

**Authors:** Michael O'Sullivan, William K. Smith, Stephen Sitch, Pierre Friedlingstein, Vivek K. Arora, Vanessa Haverd, Atul K. Jain, Etsushi Kato, Markus Kautz, Danica Lombardozzi, Julia E. M. S. Nabel, Hanqin Tian, Nicolas Vuichard, Andy Wiltshire, Dan Zhu, Wolfgang Buermann

**Affiliations:** ^1^ College of Engineering, Mathematics and Physical Sciences University of Exeter Exeter UK; ^2^ School of Natural Resources and the Environment University of Arizona Tucson AZ USA; ^3^ College of Life and Environmental Sciences University of Exeter Exeter UK; ^4^ LMD/IPSL, ENS, PSL Université, École Polytechnique, Institut Polytechnique de Paris, Sorbonne Université, CNRS Paris France; ^5^ Canadian Centre for Climate Modelling and Analysis, Environment and Climate Change Canada University of Victoria Victoria British Columbia Canada; ^6^ CSIRO Oceans and Atmosphere Canberra ACT Australia; ^7^ Department of Atmospheric Sciences University of Illinois Urbana IL USA; ^8^ Institute of Applied Energy (IAE) Minato Japan; ^9^ Institute of Meteorology and Climate Research – Atmospheric Environmental Research (IMK‐IFU) Karlsruhe Institute of Technology (KIT) Garmisch‐Partenkirchen Germany; ^10^ Forest Research Institute Baden‐Württemberg Freiburg Germany; ^11^ Climate and Global Dynamics Division National Center for Atmospheric Research Boulder CO USA; ^12^ Max Planck Institute for Meteorology Hamburg Germany; ^13^ International Center for Climate and Global Change Research, School of Forestry and Wildlife Sciences Auburn University Auburn AL USA; ^14^ Laboratoire des Sciences du Climat et de l'Environnement, UMR8212 CEA‐CNRS‐UVSQ, Université Paris‐Saclay, IPSL Gif‐sur‐Yvette France; ^15^ Met Office Hadley Centre Exeter UK; ^16^ Institute of Geography Augsburg University Augsburg Germany; ^17^ Institute of the Environment and Sustainability University of California, Los Angeles Los Angeles CA USA

**Keywords:** gross primary productivity, interannual variability, trends, climate, carbon

## Abstract

Variability in climate exerts a strong influence on vegetation productivity (gross primary productivity; GPP), and therefore has a large impact on the land carbon sink. However, no direct observations of global GPP exist, and estimates rely on models that are constrained by observations at various spatial and temporal scales. Here, we assess the consistency in GPP from global products which extend for more than three decades; two observation‐based approaches, the upscaling of FLUXNET site observations (FLUXCOM) and a remote sensing derived light use efficiency model (RS‐LUE), and from a suite of terrestrial biosphere models (TRENDYv6). At local scales, we find high correlations in annual GPP among the products, with exceptions in tropical and high northern latitudes. On longer time scales, the products agree on the direction of trends over 58% of the land, with large increases across northern latitudes driven by warming trends. Further, tropical regions exhibit the largest interannual variability in GPP, with both rainforests and savannas contributing substantially. Variability in savanna GPP is likely predominantly driven by water availability, although temperature could play a role via soil moisture‐atmosphere feedbacks. There is, however, no consensus on the magnitude and driver of variability of tropical forests, which suggest uncertainties in process representations and underlying observations remain. These results emphasize the need for more direct long‐term observations of GPP along with an extension of in situ networks in underrepresented regions (e.g., tropical forests). Such capabilities would support efforts to better validate relevant processes in models, to more accurately estimate GPP.

## Key Points

- Changes in climate over the period 1982–2016 have increased GPP at a global scale and across northern latitudes
- Savannas and tropical forests are hotspots for IAV in GPP, although the dominant climate drivers are not consistent among the assessed products
- DGVMs systematically underestimate the IAV of GPP in tropical forests, highlighting the need for improved parameterizations/formulations

## 1 Introduction

Gross primary production (GPP), the amount of carbon fixed by photosynthesis per unit area in time, is the pathway for transferring atmospheric CO_2_ to the terrestrial biosphere. It is the largest carbon flux in the Earth system, and as such any small change can significantly alter the net carbon balance at the surface, atmospheric CO_2_ concentrations, and subsequently feedbacks to climate (Friedlingstein et al., 2014). Therefore, quantifying variations in GPP and attributing underlying drivers and mechanisms is an important area of research. GPP responds to changes in atmospheric CO_2_ concentrations, nutrient availability, and climate (Ciais et al., 2005; Nemani et al., 2003; Schimel, Stephens, et al., 2015; Zaehle & Dalmonech, 2011). Moreover, land use and land cover change (e.g., deforestation) alter the spatial distribution of vegetation and therefore also impact GPP and carbon uptake.

Variations in climate can influence both interannual variability (IAV) and long‐term trends in GPP. IAV in global GPP has been found to be controlled by certain hotspot regions, specifically tropical forests (Jung et al., 2011; Wang et al., 2013, 2014) and semiarid regions (Ahlstrom et al., 2015; Poulter et al., 2014). IAV in GPP in these hotspot regions is dominated by climate variability associated with the El Niño–Southern Oscillation (ENSO) (Ahlstrom et al., 2015; Bastos et al., 2013; Zhao & Running, 2010), and other extreme climatic events (Zscheischler et al., 2014). With more frequent climate extremes projected under climate change (Seneviratne et al., 2012), the impact of GPP IAV on the global carbon cycle is also likely to increase (Reichstein et al., 2013).

In regard to longer‐term variations, there is evidence that recent large‐scale climatic shifts have profoundly influenced global plant carbon uptake and the land carbon sink (Buermann et al., 2016). Specifically, the accelerated warming over northern latitudes appears to have substantially increased carbon uptake by plants (Keenan et al., 2014; Nemani et al., 2003; Piao et al., 2007). However, warming during colder seasons in the northern continents can lead to moisture stress later in the year, offsetting the initial positive effects (Buermann et al., 2013, 2018; Lian et al., 2020). Warming‐induced drying trends with adverse impacts on GPP have also been identified in large regions of the Southern Hemisphere (Huang et al., 2016; Zhao & Running, 2010; Zscheischler et al., 2014).

Presently, large‐scale GPP estimates (including those mentioned above) can only be obtained through modeling approaches. This is because observation‐based estimates of GPP are only possible at leaf levels through chambers (Welp et al., 2011) or derived at local landscape scale through eddy covariance flux towers (Baldocchi et al., 2001). In addition, scaling leaf‐level observations to global scale is challenging due to the artificial nature of lab experiments and challenges associated with scaling leaf‐level values to the entire canopy (Baldocchi, 2003). Both the partitioning of eddy covariance net flux data into the GPP component flux (Reichstein et al., 2005; Wehr et al., 2016) and the upscaling of the eddy flux tower data to global scale introduces additional uncertainties (Beer et al., 2010; Jung et al., 2009). In an attempt to quantify such uncertainties, the FLUXCOM initiative uses a variety of methods (including different partitioning methods and machine learning algorithms) to integrate local tower observations, satellite remote sensing, and meteorological data to produce wall‐to‐wall estimates of carbon fluxes (Jung et al., 2017; Tramontana et al., 2016).

An alternative data‐driven approach to estimate GPP is using satellite‐based metrics of vegetation activity, such as the fraction of absorbed photosynthetic active radiation absorbed by plants (FPAR), combined with a light use efficiency formulation (Running et al., 2004; Zhu et al., 2013). Such satellite‐driven production efficiency models can provide high spatiotemporal information owing to the satellites unique sampling capabilities. However, satellite observations often suffer from data contamination due to cloud cover and signal saturation in dense canopied regions and these influences together with uncertainties in light‐use efficiency (LUE) theory propagate into uncertainties in GPP (Kolby Smith et al., 2016).

Dynamic global vegetation models (DGVMs) offer another method of estimating global GPP. These prognostic process‐based models represent our current understanding of the major processes of the terrestrial carbon cycle and other biogeochemical cycles (Fisher et al., 2014) and offer the only way to project future changes in carbon fluxes between land and the atmosphere and different terrestrial carbon pools. However, given the complexity of terrestrial ecosystems, simplifications must be made, and often such simplifying assumptions are different and sometimes divergent across models (Sitch et al., 2015). More specifically, differences arise between modeled estimates of GPP due to different sets of equations and parameterizations of terrestrial ecosystem processes, such as photosynthesis, leaf phenology, canopy scaling, and nutrient cycling (Fisher et al., 2014; Rogers et al., 2017).

These observation‐based and process‐based modeling approaches all have their own merits and limitations and they do not necessarily agree with each other on various spatial and temporal scales (Kolby Smith et al., 2016). Yet corresponding model outputs have been used independently or in conjunction with one another in multiple studies focusing on the variability of the terrestrial carbon cycle (Ballantyne et al., 2017; Beer et al., 2010; Jung et al., 2017; Zhu et al., 2016). It is thus important to assess the consistency of these “state‐of‐the‐art” GPP products across multiple temporal and spatial scales including their sensitivity to climate variability. Highlighting similarities and discrepancies in these products will provide information on the level of confidence that can be placed in these products and hence previously made inferences.

The aim of this study is to compare climate‐driven GPP estimates from an empirical approach based on eddy covariance data (FLUXCOM, Jung et al., 2017), a light use efficiency model based on satellite observations of vegetation activity (Kolby Smith et al., 2016), and a set of DGVMs from TRENDYv6 (Le Quéré et al., 2018) all driven with the same climate data (see section 2). Specifically, over the 35‐year study period 1982–2016, we investigate (i) to what extent are these climate‐driven GPP estimates consistent across multiple spatial and temporal scales, (ii) how they differ in regard to their climate sensitivities, and (iii) which ecosystems are contributing most to global GPP variability and trends.

## 2 Methods

### 2.1 GPP Data Sets

#### 2.1.1 FLUXCOM

For this study, we used GPP data from FLUXCOM (version RS + METEO) (Jung et al., 2017; Tramontana et al., 2016) at 0.5° spatial resolution and monthly time scale over the period 1982–2016. FLUXCOM GPP is based on machine learning methods that upscale FLUXNET (Baldocchi et al., 2001) observations of carbon fluxes, based on local meteorology derived from flux towers, using gridded climate and satellite data. Three machine learning methods are used for the upscaling process; artificial neural networks, random forests, and multivariate adaptive regression (Jung et al., 2017; Tramontana et al., 2016). Eddy covariance flux towers measure net carbon exchange between land and the atmosphere. The component GPP flux is derived by estimating the temperature sensitivity of ecosystem respiration (TER) from nighttime flux data and then extrapolated to daytime to determine TER and GPP (Reichstein et al., 2005). Gridded (0.5°) predictor variables (e.g., local climate, vegetation type, normalized difference vegetation index, NDVI) are used to produce spatiotemporal grids of GPP. Climate variables are from the CRUNCEPv8 (https://vesg.ipsl.upmc.fr/thredds/catalog/work/p529viov/cruncep/V8_1901_2016/catalog.html) product, which is based on a combination of Climate Research Unit monthly 0.5° data set and the 6‐hourly time resolution National Centers for Environmental Prediction (NCEP) reanalysis. Overall, there are three different GPP estimates available (three upscaling products with different machine learning algorithms to form the FLUXCOM ensemble) and the spread in these ensemble members is used as a measure of uncertainty (see below). By design, the FLUXCOM RS + METEO GPP product does not capture the effects associated with CO_2_ fertilization, vegetation greening, or disturbances since it is based on only time‐varying climatic input variables, and a climatological mean seasonal cycle of plant growth derived from satellite data (Tramontana et al., 2016). The FLUXCOM product therefore largely captures the response of GPP to instantaneous climate variability alone and does not capture the effect of current vegetation state, which is influenced by concurrent climate and past vegetation growth, whereby the dependency of current vegetation state on past growth is referred to as the vegetation memory effect.

In a supplementary sensitivity analysis, we also use another GPP product (0.5° over the period 1982–2008) based on upscaled FLUXNET observations (FluxNetG; Jung et al., 2011). The FluxNetG GPP product is based on both time‐varying (seasonal and interannual) satellite vegetation data (NDVIg, see Buermann et al., 2013) and climate data. A comparison of the FluxNetG and FLUXCOM GPP data therefore allows us to assess the importance of ecosystem vegetation state (including memory effects that are implicitly included in FluxNetG) on IAV and trends in GPP.

#### 2.1.2 RS‐LUE Model

Remote sensing based LUE (RS‐LUE) models offer an alternative tool to estimate GPP fluxes (Running et al., 2004). Here we use a 35‐year satellite‐driven GPP data set (1982–2016) that is based on the MODIS GPP algorithm (Kolby Smith et al., 2016; Running et al., 2004):

(1) $$\begin{array}{c} \mathrm{GPP}=\text{FPAR}\times \mathrm{PAR}\times {\mathrm{LUE}}_{\max}\times f\left(T_{\min}\right)\times f\left(\mathrm{VPD}\right) \end{array}$$

The included satellite‐based FPAR data over this extended study period is based on the normalized difference vegetation index version 3 g data set (NDVI3g) from NOAA‐AVHRR satellites using a neural network algorithm (Zhu et al., 2013). PAR represents incoming photosynthetically active radiation. The maximum light use efficiency (*LUE*_max_), minimum temperature function ( *f* (*T*_min_)), and vapor pressure deficit function (*f* (VPD)) vary depending on biome type and use gridded, monthly mean *T*_min_ and VPD from CRUNCEPv8. This formulation assumes a temporally invariant *LUE*, and therefore it does not capture the direct effect of atmospheric CO_2_ increase on GPP (via an increase in LUE) (De Kauwe et al., 2016; Norby et al., 2005; Smith et al., 2019). Consequently, changes in GPP based on Equation 1 are largely driven by climate variability and changes in FPAR. A portion of the long‐term trends in GPP could be driven by increasing CO_2_ concentrations, via changes in satellite FPAR, although this “indirect” CO_2_ fertilization effect is estimated to be relatively small (De Kauwe et al., 2016). Further, as opposed to FLUXCOM, influences of vegetation state including vegetation memory effects are included (as annually varying FPAR is used) and therefore RS‐LUE may capture an additional source of variability. We use two sets of parameters for *LUE*_max_, *f* (*T*_min_), and *f* (VPD) to provide an estimate of uncertainty in model structure (Robinson et al., 2018; Zhao & Running, 2010). We then formed an ensemble mean of the two estimates and use the spread as a measure of uncertainty (see also below).

#### 2.1.3 Trendy

Finally, GPP data from the process‐based models for the period 1982–2016 from 12 DGVMs are obtained that participated in the TRENDYv6 multimodel intercomparison and followed a common protocol (Sitch et al., 2015). Models included in this TRENDY ensemble are CABLE (Haverd et al., 2018), CLASS‐CTEM (Melton & Arora, 2016), CLM4.5‐BGC (Oleson et al., 2013), DLEM (Tian et al., 2015), ISAM (Jain et al., 2013), LPJ‐GUESS (Smith et al., 2014), JSBACH (Reick et al., 2013), JULES (Clark et al., 2011), ORCHIDEE (Krinner et al., 2005), ORCHIDEE‐MICT (Guimberteau et al., 2018), VEGAS (Zeng et al., 2005), and VISIT (Kato et al., 2013). In order to isolate the climate‐driven GPP portion in the TRENDYv6 model runs (consistent with our study aim) the following procedure was applied. We used the “CO_2_‐only (S1)” and “Climate and CO_2_ (S2)” simulations from the full set of simulations performed for TRENDYv6. Although the simulations were run from the year 1700 onward, meteorological data were available only for the period 1901–2016. The “S1” simulation is forced with time varying atmospheric CO_2_ concentrations derived from ice cores and National Oceanic and Atmospheric Administration (NOAA) monitoring stations but meteorological data from the early twentieth century (1901–1920) (CRUNCEPv8) is used repeatedly. Consequently, the S1 simulation does not capture the response of the models to changes in climate over the historical period. To derive the desired “climate‐only” response for each model we calculated the trend over 35 years using nonlinear least squares (from the “stats” package in R, Bates & Watts, 1988) (1982–2016) for each month and grid cell in the S1 simulations. Then this trend was subtracted from the S2 simulations (the S2 simulations are run with both time varying CO_2_ concentrations and meteorological data). This preserves interannual variability (driven by climate variations) but removes the influence of rising CO_2_ concentrations over this period. We then calculated ensemble mean and standard deviation which represents uncertainty based on spread across 12 participating models (see below). The “climate‐driven” TRENDY GPP product is used in all interannual and trend analyses. It is important to note that both simulations (S1 and S2) use a fixed preindustrial land cover distribution and therefore the climate‐driven GPP product derived here will not include the effects of any recent land use and land cover changes (LULCC). This contrasts with the RS‐LUE product, which will capture LULCC effects indirectly via satellite‐derived FPAR (see Equation 1). However, LULCC is not a focus of this study and it appears to not be a significant driver of GPP changes over the study period (Figures S1 and S2 in the supporting information).

### 2.2 SIF

To validate the patterns of IAV in the GPP products, we use solar‐induced fluorescence (SIF) data from the Global Ozone Monitoring Experiment (GOME‐2) aboard the MetOP‐A satellite (Joiner et al., 2013). We use the monthly mean SIF from the Level 3 data product (https://acd‐ext.gsfc.nasa.gov/People/Joiner/my_gifs/GOME_F/GOME‐F.htm). The data are provided globally at a 0.5° resolution and extends over the period 2008–2016. We first create annual means and then detrend (via linear regression) the data to remove any effects from long‐term changes and also because we focus on analyzing patterns of IAV.

### 2.3 Climate Data Sets

We calculate the sensitivity of each GPP data set to surface air temperature, precipitation, and solar radiation obtained from the CRUNCEPv8 reanalysis at 0.5° resolution. All three global GPP products that are evaluated in this study are driven by surface air temperature and incoming solar radiation; however, only TRENDYv6 GPP simulations are directly driven with precipitation. In the FLUXCOM approach, moisture limitations are represented through a water stress function based on a soil water balance model (Tramontana et al., 2016), whereas in the RS‐LUE formulation a VPD scalar is used (see Equation 1 and, e.g., Yuan et al., 2019). Precipitation is a crucial parameter influencing VPD and soil moisture, and thus we use it consistently across data sets to estimate corresponding GPP sensitivities to water availability. We also note that there are uncertainties associated with climate reanalyses, in particular with tropical solar radiation (Wu et al., 2018) and our model results are highly dependent on the climate forcing we have used. However, CRUNCEP outperforms other commonly used climate data sets and has been shown to work better in tropical regions for simulating GPP (Wu et al., 2018). Further, as all the products are driven with the same climate forcing, differences among models are due to structural differences in the respective approaches for estimating GPP.

### 2.4 Data Processing

The FLUXCOM and RS‐LUE GPP products have a native spatial resolution of 0.5° × 0.5°, and for consistency we regridded the DGVM‐based GPP data to match this spatial scale. All products are available on monthly time steps from 1982–2016. For each ensemble member (3 for FLUXCOM, 12 for TRENDY, and 2 for RS‐LUE) we calculated local (grid cell level) and regional (see Figure S3 for regional definitions) anomalies by subtracting the 35‐year mean at monthly, seasonal, and annual time scales. We then created ensemble means of these anomalies for each product and use the spread (1 stdev) as a measure of uncertainty in the various approaches. We focus on anomalies rather than absolute GPP as there are large differences in the magnitudes of GPP estimates across all 17 data sets, with annual mean values ranging from 90 to 200 PgC/yr (Figure S4).

### 2.5 Statistical Analysis

In order to assess the sensitivity of the various GPP products to climate, we decomposed GPP anomalies for each region (*r*) and time period (*s*) into the components forced by temperature, precipitation, and radiation as

(2) $$\begin{array}{c} {\mathrm{GPP}}_{r,s}=\gamma_{r}\times {\text{TEMP}}_{r,s}+\lambda_{r}\times {\text{PREC}}_{r,s}+\delta_{r}\times {\mathrm{RAD}}_{r,s}+\varepsilon_{r,s} \end{array}$$

where *γ*_*r*_ represents the sensitivity of GPP to temperature anomalies (*TEMP*_*r*,*y*_), *λ*_*r*_ the sensitivity of GPP to precipitation anomalies (*PREC*_*r*,*y*_), *δ*_*r*_ the sensitivity of GPP to radiation anomalies (*RAD*_*r*,*y*_), and *ε*_*r*,*y*_ is the residual error (Piao et al., 2013). For the IAV analysis, we performed this regression on detrended data (linear trend removed). Importantly, the fitted regression coefficients (*γ*_*r*_, *λ*_*r*_, and *δ*_*r*_) represent apparent GPP sensitivities to variations in temperature, precipitation, and radiation. The regression coefficients are calculated using linear least squares and uncertainty in the coefficients is obtained from the standard error. Further, GPP sensitivity to climatic drivers may change over time and in our analysis, they represent mean sensitivities over the 35‐year period. To derive the relative importance of the three climate regressors in determining GPP IAV, we use the Lindeman‐Merenda‐Gold (LMG) method (from the “relaimpo” package in R; Grömping, 2006) which calculates the contribution of each regressor to the overall R^2^ of the linear model.

For trend analysis, we first calculate linear trends for each ensemble member and from them calculate the ensemble mean trend. Uncertainty associated with the trend is calculated from the spread in trends among ensemble members. To isolate the contribution of each climate forcing to the 35‐year trend, we performed the regression (Equation 2) on the nondetrended data and then calculated the linear trend in the reconstructed GPP time series for each climate variable. For example, to calculate the trend in GPP due to temperature, we first reconstruct the GPP time series for each climate variable (from Equation 2) and then calculate the temporal trend (over the 35‐year period) in *γ*_*r*_ × *TEMP*_*r*,*s*_. Finally, we normalize the trend for each of the three products by the mean of the absolute trends across all vegetated grid cells as we focus more on spatial patterns and the underlying driving factors.

## 3 Results

### 3.1 Large‐Scale Trends and IAV in GPP

**Figure 1:**
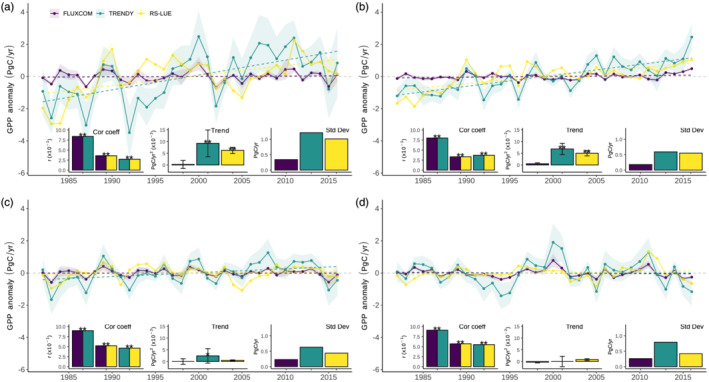
Global and regional variations in annual GPP based on three GPP products. Annual GPP anomalies (PgC/yr) over the period 1982–2016 estimated by upscaled flux tower observations, FLUXCOM (purple), a set of terrestrial biosphere models from TRENDYv6 intercomparison (teal), and a satellite‐based light use efficiency model (yellow). GPP anomalies are shown for (a) Global, (b) Northern, (c) Tropical, and (d) Southern regions, as defined in Figure S1 in the supporting information. Shading represents 1σ spread among each products ensemble members (see section 2). Linear trends are depicted with a dashed line. Bar charts (from left to right) show the correlation (Cor coeff) between detrended annual mean GPP for each product combination, the linear trend (PgC/yr2) in annual mean GPP for each product (error bars represent uncertainty defined as the standard deviation of the trends of each group of ensemble members), and the interannual variability of each product as the 1σ (PgC/yr) of the detrended time series. Trends significantly different from zero and significant correlations are marked with an asterisk (*P < 0.05, **P < 0.01).

In a first step, we compared trends and IAV in climate‐driven GPP based on the three global products, FLUXCOM, TRENDYv6, and RS‐LUE at large spatial scales. At global scale, all three GPP products show a positive trend over the study period 1982–2016 (Figure 1a). However, the FLUXCOM GPP data show a substantially smaller nonsignificant increase (0.002 ± 0.017 PgC/yr^2^; *P* > 0.05) compared to TRENDYv6 (0.092 ± 0.057 PgC/yr^2^; *P* < 0.01) and RS‐LUE (0.062 ± 0.022 PgC/yr^2^; *P* < 0.01) (Figure 1a). We also found that individual FLUXCOM ensemble members do not agree on the direction of the GPP trend (Figure S5), hence the large uncertainty in estimates based on the ensemble mean. This highlights the influence of the choice of upscaling method on GPP estimates (Tramontana et al., 2016).

A focus on regional scales shows that the robust positive global trends in GPP for TRENDYv6 and RS‐LUE can be mainly attributed to significant (*P* < 0.01) increases in GPP in the northern latitudes, whereas no substantial increases in GPP are evident for FLUXCOM (Figure 1b). In the tropics, TRENDYv6 GPP data show a robust (*P* < 0.05) positive trend, whereas RS‐LUE and FLUXCOM show no significant change (Figure 1c). In the southern extratropics, none of the three products exhibits a significant trend in GPP (Figure 1d). Importantly, individual ensemble members of all three products disagree on the direction of trend in the tropics and for TRENDYv6 also in the southern latitudes (Figure S5), highlighting large uncertainties specifically in tropical GPP estimates.

Differences in the IAV between the three GPP products (estimated through the standard deviation (σ) of annual (detrended) GPP over the period 1982–2016) are also apparent. FLUXCOM GPP displays much lower global IAV (σ = 0.34 PgC/yr) compared to the two other products, (TRENDYv6: 1.21 PgC/yr and RS‐LUE: 1.01 PgC/yr; Figure 1a). Similar differences in IAV between the three products are apparent for northern and tropical regions (Figures 1b and 1c), but for the southern extratropics TRENDYv6‐based GPP is twice as high as in the other products (Figure 1d). FLUXCOM captures only a portion of the climate‐driven GPP signal, that is the direct simultaneous response of GPP to the climatic anomalies whereas the other products also contain the influence of climate on the vegetation state (concurrent and memory effects, see also section 2). Corresponding results show that the magnitude of IAV and trends in this FluxNetG product are more comparable to TRENDYv6 and RS‐LUE in all regions (Figure S6). This, together with other studies (Buermann et al., 2018), suggests that current vegetation state, influenced by current climate and effects of past vegetation growth, have a substantial role for both IAV and trends in GPP, and the omission of such effects in the present FLUXCOM product may result in an underestimation of GPP variability and long‐term trends (Jung et al., 2020).

How consistent is the IAV in the three global GPP products? To answers this, we computed correlations between the respective detrended annual GPP time series. We find that the three GPP products are significantly (*P* < 0.01) correlated in all large spatial domains, apart from FLUXCOM and RS‐LUE over northern latitudes and TRENDYv6 and RS‐LUE at global scales (see insets in Figure 1). Generally, the agreement in IAV between FLUXCOM and TRENDYv6 at both global and regional scales is substantially higher when compared to the those based on either of these two products with RS‐LUE and the agreement in IAV between all three products tends to be also greater in tropical and southern latitudes (Figure 1). RS‐LUE GPP data exhibit a large negative tropical anomaly in 2005 (Figure 1c), in response to a severe large‐scale drought (Phillips et al., 2009; Zhao & Running, 2010), a pattern that is not captured in the other two products. This suggests that the satellite‐based RS‐LUE approach captures unique information compared to FLUXCOM and TRENDYv6. This fact together with evidence that satellite‐driven approaches generally tend to agree better with in situ data in estimating GPP than approaches without satellite data (Raczka et al., 2013) may imply that both FLUXCOM and TRENDYv6 are potentially missing important information about GPP variability.

### 3.2 Local‐Scale IAV in GPP

**Figure 2:**
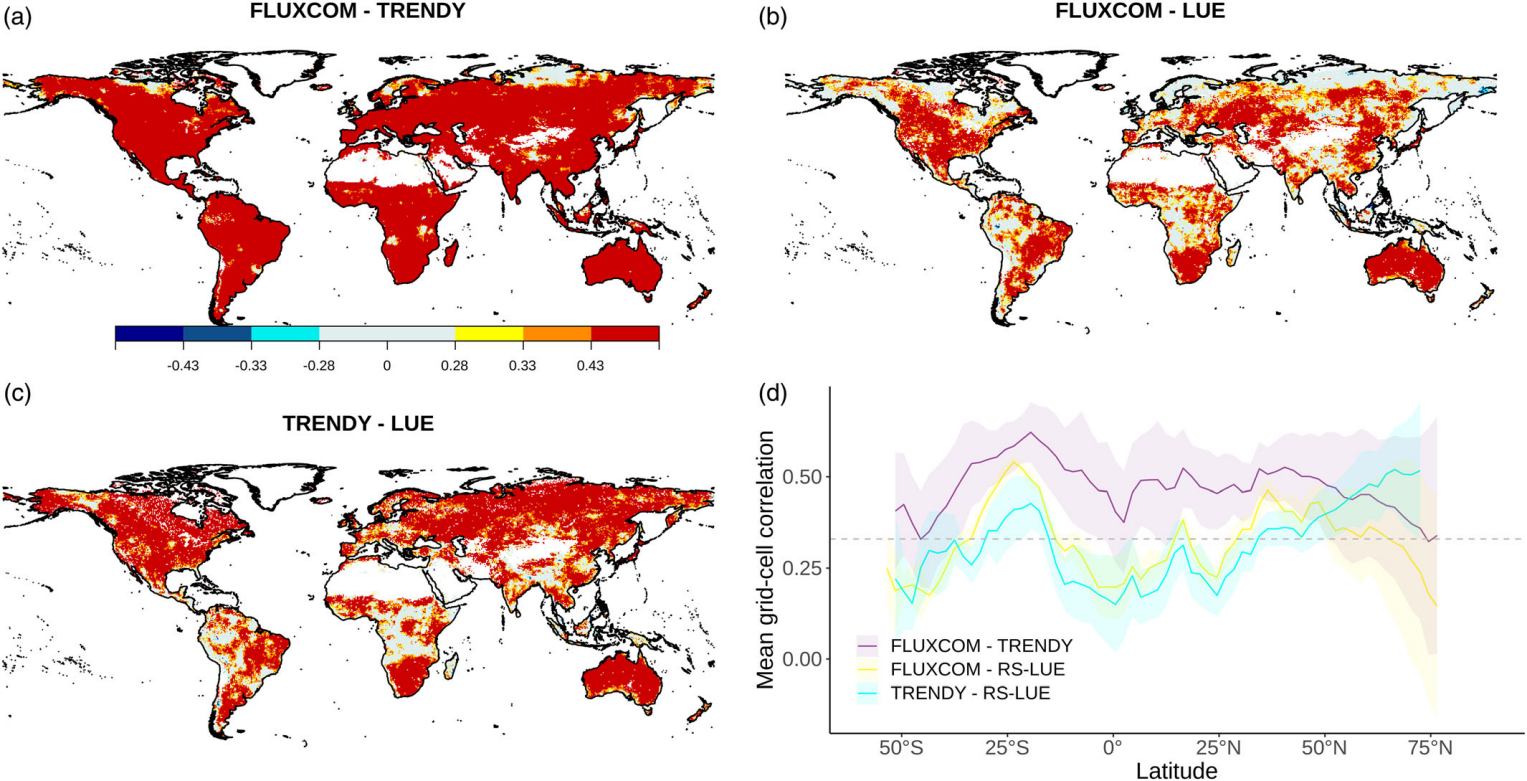
Spatially explicit consistency in IAV of GPP based on three global products. Shown are grid‐cell correlation coefficient (Pearsons' *r*) patterns between detrended annual mean GPP anomalies (1982–2016) for (a) FLUXCOM and TRENDYv6, (b) FLUXCOM and RS‐LUE, (c) TRENDY and LUE. (*r* = 0.28, 0.33, 0.43 corresponds to *P* < 0.1, *P* < 0.05, *P* < 0.01). Panel (d) shows the latitudinal distribution of correlations for each product combination. Shading represents the 1 sd spread for each product ensemble combination. Gray dashed line at *r* = 0.33 (*P* < 0.05).

We further examined the temporal agreement between data sets at more local scales by performing grid cell correlations between the detrended GPP time series. We identified large areas of significant (*P* < 0.05) positive correlations between the products (Figure 2), specifically across the temperate United States, the grasslands of Eurasia, and over the savannas and shrublands of the Southern Hemisphere (as classified by the MODIS land cover product MCD12C1 (Friedl et al., 2010), see also Figure S7). Furthermore, for FLUXCOM and TRENDYv6, significant positive correlations are found across the globe (Figure 2a). Throughout the tropics, both FLUXCOM and TRENDYv6 GPP show relatively low agreement in IAV with GPP derived from RS‐LUE. Differences also exist in boreal regions, where IAV in FLUXCOM and RS‐LUE GPP show low agreement, but TRENDYv6 and RS‐LUE are ubiquitously significantly positively correlated. With a focus on seasonal time scales, the good agreement in the IAV of GPP based on FLUXCOM and TRENDYv6 is evident for all seasons (Figure S8). For TRENDYv6 and RS‐LUE, strong positive correlations in the extratropics are apparent in all seasons, matching the annual correlation pattern (Figure 2). However, the seasonal patterns for FLUXCOM and RS‐LUE differ from the annual correlations. In the high northern latitudes, for example, the GPP of each product combination in boreal spring is positively correlated, whereas there are no significant correlations in boreal summer for the combination FLUXCOM and RS‐LUE (Figure S8). The causes of the seasonal GPP discrepancies in these two products are difficult to diagnose, but may result from structural differences in the products (e.g., photosynthetic and/or phenological parameterization, land cover parameterization, method of including climatic constraints), or, for example, for high northern latitudes biases in satellite data (Guay et al., 2014) and lack of eddy covariance flux towers in these remote regions.

**Figure 3:**
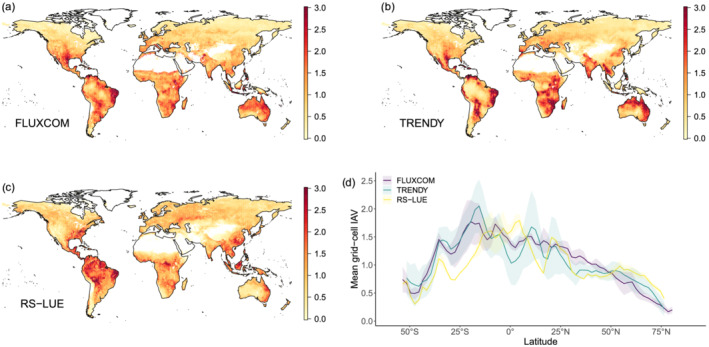
Hotspots of interannual variability in GPP. Maps of the relative magnitude of interannual variability in annual mean GPP over the period 1982–2016 defined for each pixel as the standard deviation in annual mean GPP divided by the mean standard deviation of all grid cells. (a) FLUXCOM, (b) TRENDY, (c) RS‐LUE. Panel (d) shows the latitudinal distribution of relative (normalized by global mean) deviations for the three products.

We next assessed the consistency between the three products in regard to the spatial distribution of “hotspots” of IAV in GPP. Based on our results (see above) and indicated previously (Jung et al., 2017), FLUXCOM GPP IAV appears to be systematically too small, with mean grid cell variations (defined as the mean of all vegetated grid cell standard deviations of detrended annual mean GPP) of 26 g C/m^2^/yr compared to TRENDYv6 and RS‐LUE with mean grid cell variations of 96 and 41 g C/m^2^/yr, respectively (Figure S9). To make the three data sets more comparable, we focus predominantly on the spatial patterns and give less emphasis to absolute values. Figure 3 depicts the geographic distribution of the relative standard deviation (normalized by the global mean grid‐cell standard deviation) in annual mean GPP. Interannual variability in GPP is not uniformly distributed across the globe, with certain regions dominating the signal. For example, Southern North America, Northeastern Brazil, Southern Africa, and the Eastern Australia are clear hotspots of GPP IAV in all three products. These regions are dominated by short vegetation types (shrublands and savannas; Figure S7) which have been previously shown to heavily influence global GPP IAV (Ahlstrom et al., 2015; Poulter et al., 2014; Zscheischler et al., 2014). Both FLUXCOM and TRENDYv6 have generally similar patterns globally, including large regions of Southern USA/Mexico and Eastern Africa. Interestingly, for RS‐LUE (and FLUXCOM to a lesser extent), South American and Southeast Asian tropical forests exhibit relatively high IAV in GPP, a pattern missing in TRENDYv6 (Figures 3 and S10). Interestingly, RS‐LUE and FLUXCOM have low temporal agreement in tropical forests, indicating variability in these two products is driven by different processes. It is worth noting that tropical regions are not well covered by FLUXNET sites, leading to large uncertainties in the FLUXCOM estimate. Further, the general lack of in situ observations in tropical latitudes (Cleveland et al., 2015; Schimel, Pavlick, et al., 2015), limits a realistic presentation of photosynthesis in DGVMs, impacting the TRENDYv6 GPP estimates also.

**Figure 4:**
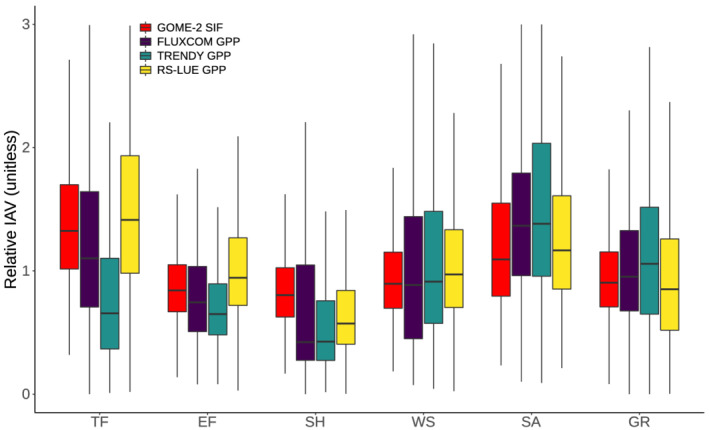
The contribution of different vegetation types to the IAV in vegetation productivity. The plot shows the relative magnitude of IAV (using standard deviation as a measure of IAV) in (detrended) annual mean SIF (GOME‐2) and GPP across biomes (Figure S7) over the period 2008–2016. Boxplots show the grid cell level variability and are generated by first calculating the standard deviation of (detrended) annual mean SIF or GPP for all grid cells, then grouping grid cells together by land cover type, and finally normalizing by the global mean grid cell standard deviation for each product separately.

In order to test the robustness of the IAV in GPP based on the three assessed products, and elucidate the role of tropical forests in particular, we compared GPP IAV to those based on an independent satellite‐based SIF data set (Joiner et al., 2013) (a proxy of photosynthesis; Frankenberg et al., 2011). At biome level, we found that both tropical forest and savanna ecosystems have relatively high IAV in annual mean SIF, corroborating the results for RS‐LUE and FLUXCOM (Figure 4). A comparison at finer spatial scales (e.g., grid cell correlations) is limited due to the inherent noise of the SIF data at interannual time scales (Butterfield et al., 2020).

### 3.3 Climate Sensitivities

**Figure 5:**
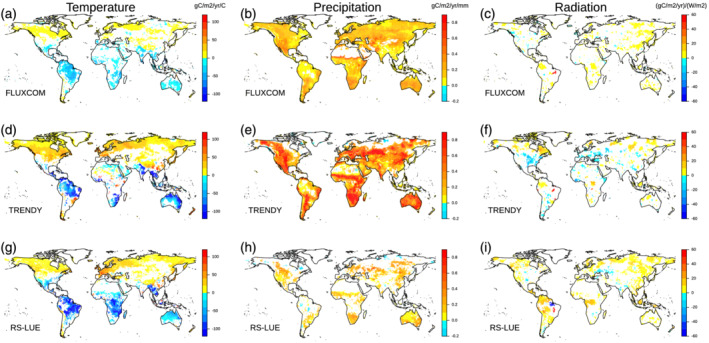
Spatial patterns of the sensitivity of GPP to variability in (a, d, g) mean annual temperature (gC/m^2^/yr/°C), (b, e, h) mean annual precipitation (gC/m^2^/yr/mm), and (c, f, i) mean annual solar radiation ([gC/m^2^/yr]/[W/m^2^]) for (a, b, c) FLUXCOM, (d, e, f) TRENDYv6, and (g, h, i) RS‐LUE. Sensitivities are calculated from multiple linear regression of annual GPP against temperature, precipitation, and radiation at each grid cell (see Equation 2 in section 2). Only significant (*P* < 0.05) sensitivities are shown.

We further calculated GPP sensitivities to mean annual temperature, precipitation, and solar radiation at local scales using our regression framework (see Equation 2 in section 2). The geographic distribution for annual GPP against temperature is similar across the three products, with a positive relationship in Eurasia and North America and a negative relationship in the tropics and southern latitudes (Figures 5 and S11). For FLUXCOM, GPP response to temperature is much lower than for TRENDYv6 and RS‐LUE and corresponding significant (*P* < 0.05) sensitivities are not as widespread (35% of vegetated northern land (>30°N) for FLUXCOM compared to 65% for TRENDYv6 and 61% for RS‐LUE). All products exhibit a similar spatial pattern of significant negative GPP responses to temperature across large parts of South America, Southern Asia, and Australia (Figure 5). Regarding GPP responses to precipitation anomalies, the three products agree on significant positive sensitivities in the northern midlatitudes (30–50°N), and the savannas and shrublands of South America, South Africa, and Australia (Figures 5 and S11). In general, however, RS‐LUE GPP exhibits much fewer significant correlations with precipitation outside of arid regions compared to FLUXCOM and TRENDYv6. Further, significant sensitivities of GPP to solar radiation are not as extensive (compared to temperature and precipitation) in all three products, except for RS‐LUE where tropical and high northern latitudes show clear positive correlations between GPP and radiation.

**Figure 6:**
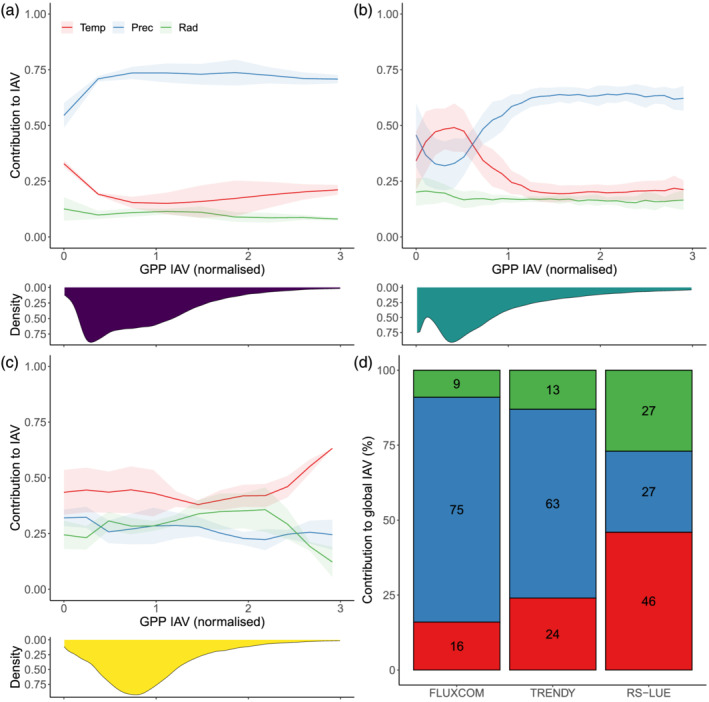
Relationship between IAV in GPP and climate variables. Shown is the contribution of each climate variable to the IAV in GPP across a range of GPP variabilities (normalized by the mean grid cell IAV for each product) for (a) FLUXCOM, (b) TRENDY, (c) RS‐LUE. Shading represents the uncertainty as defined by the standard deviation across ensemble members after subtracting the mean contribution across the whole GPP IAV range. Density plots represent the distribution in grid cell IAV values. By combining the individual climate contributions with the magnitude of GPP IAV and the IAV magnitude distributions, we can calculate the contribution (%) of each climate factor to global IAV in GPP, shown in panel (d).

As a next step, we combined our previously calculated climate sensitivities with the magnitude of IAV in GPP on grid cell scales in order to assess the contribution of each climate variable to the overall variability. We then integrated the local‐scale information to quantify the contribution of temperature, precipitation, and radiation to global variability in GPP. We found that for the FLUXCOM and TRENDYv6 products, precipitation is the dominant driver of GPP IAV (at global scale and across the majority of the land surface), albeit with large regional variations in the magnitude of dominance depending on the underlying vegetation type (Figures 6 and S11). In contrast, RS‐LUE‐based GPP IAV is predominantly driven by temperature across the whole range of variability and all global biomes (dominant over 46% of the vegetated land surface), with about equal contributions from precipitation and radiation (Figures 6 and S11). The primary difference between FLUXCOM and TRENDYv6 originates from contrasting GPP sensitivity patterns to precipitation and temperature in regions of relatively low IAV: extratropical forests and shrublands. In comparison to FLUXCOM, where GPP IAV is primarily controlled by precipitation, TRENDYv6 GPP IAV is substantially more influenced by temperature. Further differences exist across the tropical latitudes, with FLUXCOM and TRENDYv6 suggesting that tropical forest GPP IAV is controlled mainly by precipitation and temperature, whereas the RS‐LUE product indicates radiation plays a more important role, particularly in wet tropical forests (Figures 5 and S11). The savannas of South America and Africa, and much of southern latitudes are known to be water‐limited regions (Humphrey et al., 2018; Jung et al., 2017; Nemani et al., 2003), giving confidence in FLUXCOM and TRENDYv6 sensitivity patterns. In contrast, RS‐LUE GPP IAV in these regions is more controlled by temperature. This divergence in precipitation sensitivity in RS‐LUE GPP is likely partially impacted by the RS‐LUE algorithm formulation, which includes a VPD‐based biophysical constraint, but lacks a soil moisture‐based biophysical constraint, resulting in a more pronounced and potentially exaggerated sensitivity to temperature. It is important to note that there is a strong seasonal dependency of the GPP climate sensitivities, for example, for extratropical forests and shrublands temperature controlling spring (MAM) GPP IAV and precipitation driving GPP variability in summer (JJA) months (Figure S12) relatively consistently in all three products.

### 3.4 Local‐Scale Trends in GPP

**Figure 7:**
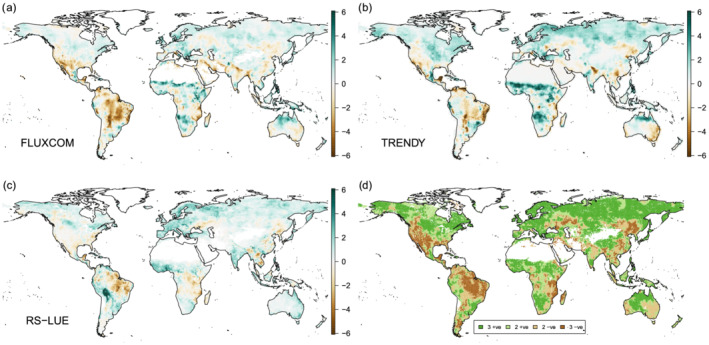
Spatial pattern of climate‐driven trends in annual mean GPP based on three products. (a, b, c) The maps depict relative (normalized by the globally integrated GPP trend) trends in annual GPP over 1982–2016 for (a) FLUXCOM, (b) TRENDYv6, (c) RS‐LUE. (d) Agreement in the direction of trend between the three products. Dark areas are where all agree on the direction of trend and light areas indicate disagreement (two products agree, and one differs).

An ensuing trend analysis on annual GPP shows that all three products exhibit consistently positive trends for large areas across Boreal Eurasia, Europe, North America, West and South Africa, and Australia (Figure 7). However, only TRENDYv6 and RS‐LUE indicate large‐scale increases for the northern latitudes, whereas in the case of FLUXCOM such widespread positive trends are less frequent. For both TRENDYv6 and RS‐LUE, extratropical forests and shrubland annual GPP increases arise largely from a positive response to temperature in spring (MAM) and summer (JJA) months, respectively (Figures S13 and S14) indicating enhanced photosynthesis within the growing season and/or a lengthening the growing season (Forkel et al., 2016; Keenan et al., 2014). Interestingly, in the boreal summer months (JJA), the warming trend had a negative impact upon FLUXCOM and RS‐LUE extratropical forest GPP but a positive impact for TRENDYv6 (Figure S14). This may suggest that the use of preindustrial land cover in the TRENDYv6 product leads to errors (e.g., too much forest) or current land surface models are potentially missing important processes, such as the buildup of water stress due to warmer springs (Buermann et al., 2018; Wolf et al., 2016), or an increase in the number of extreme warm days reducing vegetation productivity (Wang et al., 2018). Furthermore, in contrast to TRENDYv6 and RS‐LUE, FLUXCOM shrubland GPP shows no response to increased temperatures (Figures S13 and S14). Northern latitude shrublands are largely not covered by FLUXNET towers suggesting that these regions and corresponding climate links are not well captured in the FLUXCOM product (Tramontana et al., 2016) and uncertainties are likely large.

The largest relative (positive) GPP trends which are also consistent in all three products are in the savannas of Southern and West Africa, owing to increased precipitation trends (Figures 7 and S13). Southern United States (shrublands), South America (tropical forest and savannas), East Africa (savannas), and North East China (grasslands) exhibit a declining trend in GPP in all data sets (Figure 7). Warmer temperatures in these regions (Figure S15) could have led to increases in evaporative demand, leading to a reduction in photosynthesis (e.g., Zhao & Running, 2010). Moreover, the negative response of tropical GPP to increasing temperatures that is also consistently seen in all three products may imply that tropical ecosystems are already functioning near their temperature optimum and any further increases in temperature will negatively affect their GPP (Corlett, 2011).

Overall, we find that the three products agree in regard to the direction of trends over 58% of the vegetated land surface (45% and 13% for positive and negative trends, respectively) (Figure 7d). In addition, when integrated to the global scale, FLUXCOM and TRENDYv6 GPP trends are predominantly driven by temperature trends (48% and 53%, respectively; Figure S13), with precipitation trends also having an important role (42% and 36%, respectively). However, RS‐LUE globally integrated GPP trend is more driven by temperature and radiation (69% and 19%) with a minor role for precipitation.

## 4 Discussion

Understanding and quantifying how photosynthesis responds to past climate change is a necessary step toward improved process understanding and more robust future climate projections. Analyzing similarities and differences between three commonly used independent “state‐of‐the‐art” data sets enables the formation of cogent conclusions about climate impacts on productivity and also reveals key knowledge gaps for the community to address. Our study unraveled both consistent and inconsistent patterns in trends and IAV as well as climate sensitivities in GPP across various spatial scales in the three products as well as in their climate sensitivities.

Our analyses show that TRENDYv6 and RS‐LUE GPP data exhibit a significant climate‐induced increase in global GPP over our study period, primarily driven by widespread increases across northern latitudes, a result in line with satellite, and in situ studies (e.g., Keenan et al., 2014; Myers‐Smith et al., 2015; Xu et al., 2013). Conversely, we find that FLUXCOM GPP had no significant changes (on global or broad regional scales), implying FLUXCOM GPP should be used with caution for trend analysis, as indicated previously (Tramontana et al., 2016). FLUXCOM is known to perform poorly in northern high latitudes due to the low sampling of FLUXNET sites in cold/dry climate space (Tramontana et al., 2016). Further, by design, FLUXCOM does not incorporate information on current vegetation state, and so neglects potential effects of past and concurrent vegetation growth on GPP. The FLUXCOM‐based results contrast with our analysis of a different version of upscaled flux tower GPP data (FluxNetG), which show significantly larger IAV and trends that are more consistent with those based on TRENDYv6 and RS‐LUE (see Figure S6). This finding, in line with evidence from previous studies (Besnard et al., 2019; Jung et al., 2020), suggests that to accurately estimate IAV in plant carbon uptake, it is essential to include temporally evolving information on ecosystem functioning including vegetation memory effects (e.g., via satellite vegetation data).

Another key finding is that all three products consistently indicate that tropical regions dominate the IAV of global GPP, with semiarid savannas being major hotspots of variability (Figure 3). A dominant contribution of semiarid ecosystems to tropical IAV in GPP is also supported by a satellite‐based SIF analysis (Figure 4) and biomass data (Fan et al., 2019). Across tropical savannas, rainfall as the dominant driver of variability in plant productivity, as captured in TRENDYv6 and FLUXCOM, appears robust since these ecosystems are highly water‐stressed for a prolonged period of the year (Beer et al., 2010; Poulter et al., 2014). Conversely, GPP IAV based on RS‐LUE over these regions is predominantly driven by variations in temperature and is less sensitive to variations in precipitation (although significant sensitivities to precipitation do exist). This apparent contradiction may be a result of the specific way by which moisture limitations in the RS‐LUE model are represented, namely through VPD exclusively (which is also dependent on temperature). Further, the RS‐LUE model also lacks a representation of soil moisture and the ability of VPD alone to fully capture the down regulation of GPP in drought conditions has been called into question (Stocker et al., 2018), although other studies emphasize the dominant role of atmospheric moisture conditions in determining plant growth (Koren et al., 2018; Novick et al., 2016; Yuan et al., 2019). Further, differences in our calculated climate sensitivities among the data sets may be also a result of the covariation of temperature and water availability due to soil moisture‐atmosphere feedbacks (Beer et al., 2010). Therefore, soil moisture effects on productivity can manifest themselves indirectly through variations in atmospheric temperature and moisture conditions (Anderegg et al., 2019; Zhou et al., 2019). Overall, high IAV in GPP over savannas appears robust among the three approaches and it is likely that the primary driver of GPP is local moisture conditions (Green, 2019; Humphrey et al., 2018; Poulter et al., 2014).

Furthermore, RS‐LUE and FLUXCOM (to a lesser degree) also suggest high IAV in GPP in tropical forests in line with our SIF‐based analysis (Figure 4). Our sensitivity analysis shows that for RS‐LUE, the IAV in tropical forest GPP is controlled by both radiation and temperature, whereas in the case of FLUXCOM radiation is less dominant (here precipitation plays a larger role). Over moist dense forested regions, it is well established that radiation can be the limiting factor for photosynthesis due to persistent cloud cover (Guan, 2015; Nemani et al., 2003), and the RS‐LUE model seems capable of capturing corresponding influences. We also find that FLUXCOM GPP generally has low temporal agreement with RS‐LUE‐based GPP in tropical forests and TRENDYv6 shows substantially smaller IAV in GPP in tropical forests. This implies that both the process‐based models and machine learning approach struggle to capture radiation‐driven variability in GPP suggesting limitations in simulating light‐limited growth dynamics in these two approaches (Restrepo‐Coupe et al., 2017). Process‐based models have been shown to not capture adequately the radiation‐driven observed seasonal changes in leaf area index (LAI) in tropical forests because leaf phenology may be represented too simplistic (e.g., some models simulate constant leaf growth and senescence over the year) (Restrepo‐Coupe et al., 2017). One potential missing process in this regard is new leaf growth during the dry season (decreased cloud cover leading to enhanced incoming radiation) increasing light use efficiency and GPP. This has been highlighted as a key driver of GPP variability on seasonal and interannual time scales (Brando et al., 2010; Wu et al., 2016).

In addition, DGVMs may also struggle to simulate the GPP response to IAV in temperature in tropical forests. For example, tropical forest canopy temperatures may exceed optimum temperatures for photosynthesis (e.g., during warm ENSO years) as suggested by empirical studies (Mau et al., 2018; Slot et al., 2016), but DGVMs may not capture this response. Alternatively, physiology‐based data indicate that the optimum temperatures for key biophysical parameters (Vcmax, Jmax) might be high (Slot & Winter, 2017), and it is the stomatal conductance response to VPD that drives the negative responses of GPP to high temperatures (Lloyd & Farquhar, 2008; Slot & Winter, 2017). However, there are structural differences (e.g., sensitivities to humidity or VPD) among stomatal conductance models and large uncertainties exist in the parameterizations which lead to a large range of photosynthetic responses to changing environmental conditions (Rogers et al., 2017). In general, large uncertainties associated with the parameterization, representation of thermal acclimation of photosynthetic parameters and respiration, and scaling (vertically through the canopy and spatially across the landscape) of photosynthesis have been highlighted (Rogers et al., 2017). Addressing these issues by including additional complexities must be done carefully since including more processes may introduce more uncertain parameterizations (Prentice et al., 2015; Zaehle et al., 2014).

## 5 Conclusions

Overall, we find increases in GPP at a global scale and many regions across the land surface, driven by large‐scale warming across shrublands and forests in northern latitudes and enhanced precipitation in tropical and southern semiarid lands. Further, tropical forests and savannas are the two ecosystems with the largest IAV in GPP, with water availability controlling savanna productivity. While observational studies are indicative of tropical forests being light‐limited, only one model (based on satellite‐driven light use efficiency) captures corresponding influences. In general, the inconsistencies in the GPP simulations in the three products unraveled here, suggest that uncertainties in both process representations and underlying observations that constrain GPP estimates remain. Therefore, there is a need for more long‐term, direct observations of GPP especially in underrepresented regions (e.g., tropical forests, savannas, high latitudes) to elucidate the precise dynamics and drivers of variability in photosynthesis and also reduce uncertainties in all GPP products.

## Supporting information

Figure S1

## Data Availability

The research data supporting this publication are openly available from the University of Exeter at this site (https://doi.org/10.24378/exe.2883).
