# Supplementary material for: Climate‐Driven Variability and Trends in Plant Productivity Over Recent Decades Based on Three Global Products
Source: Global Biogeochem Cycles. 2020 Dec 8;34(12):e2020GB006613. doi: 10.1029/2020GB006613 (PMC7757257; doi:10.1029/2020GB006613)
Supplement: Supplementary file 1 — Figure S1 [file GBC-34-e2020GB006613-s001.docx]

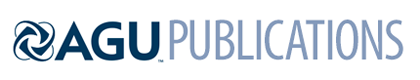


*Global Biogeochemical Cycles*

Supporting Information for

**Climate-driven variability and trends in plant productivity over recent decades based on three global products**

***Authors***

Michael O’Sullivan^1*^, William K. Smith^2^, Stephen Sitch^3^, Pierre Friedlingstein^1,4^, Vivek K. Arora^5^, Vanessa Haverd^6^, Atul K. Jain^7^, Etsushi Kato^8^, Markus Kautz^9,10^, Danica Lombardozzi^11^, Julia E.M.S. Nabel^12^, Hanqin Tian^13^, Nicolas Vuichard^14^, Andy Wiltshire^15^, Dan Zhu^14^, and Wolfgang Buermann^16,17^

^1^College of Engineering, Mathematics and Physical Sciences, University of Exeter, Exeter EX4 4QF, UK

^2^School of Natural Resources and the Environment, University of Arizona, Tucson, AZ 85721, USA

^3^College of Life and Environmental Sciences, University of Exeter, Exeter EX4 4RJ, UK

^4^LMD/IPSL, ENS, PSL Université, École Polytechnique, Institut Polytechnique de Paris, Sorbonne Université, CNRS, Paris France

^5^Canadian Centre for Climate Modelling and Analysis, Environment and Climate Change Canada, University of Victoria, Victoria, British Columbia, Canada V8W2Y2

^6^CSIRO Oceans and Atmosphere, Canberra, ACT 2601, Australia

^7^Department of Atmospheric Sciences, University of Illinois, Urbana, IL 61801, USA

^8^Institute of Applied Energy (IAE), Minato, Tokyo 105-0003, Japan

^9^Institute of Meteorology and Climate Research – Atmospheric Environmental Research (IMK-IFU), Karlsruhe Institute of Technology (KIT), 82467 Garmisch-Partenkirchen, Germany

^10^Forest Research Institute Baden-Württemberg, 79100 Freiburg, Germany

^11^Climate and Global Dynamics Division, National Center for Atmospheric Research, Boulder, CO 80302, USA

^12^Max Planck Institute for Meteorology, Hamburg 20146, Germany

^13^International Center for Climate and Global Change Research, School of Forestry and Wildlife Sciences, Auburn University, 602 Duncan Drive, Auburn, AL 36849, USA

^14^Laboratoire des Sciences du Climat et de l’Environnement, UMR8212 CEA-CNRS-UVSQ, Université Paris-Saclay, IPSL, Gif-sur-Yvette 91191, France

^15^Met Office Hadley Centre, Exeter EX1 3PB, UK

^16^Institute of Geography, Augsburg University, 86159 Augsburg, Germany

^17^Institute of the Environment and Sustainability, University of California, Los Angeles, Los Angeles, CA 90095, USA

*Corresponding author: Michael O’Sullivan

College of Engineering, Mathematics and Physical Sciences,

University of Exeter,

Exeter EX4 4QF,

United Kingdom

Email: [m.osullivan@exeter.ac.uk](mailto:m.osullivan@exeter.ac.uk)

**Contents of this file**

Figures S1 to S15


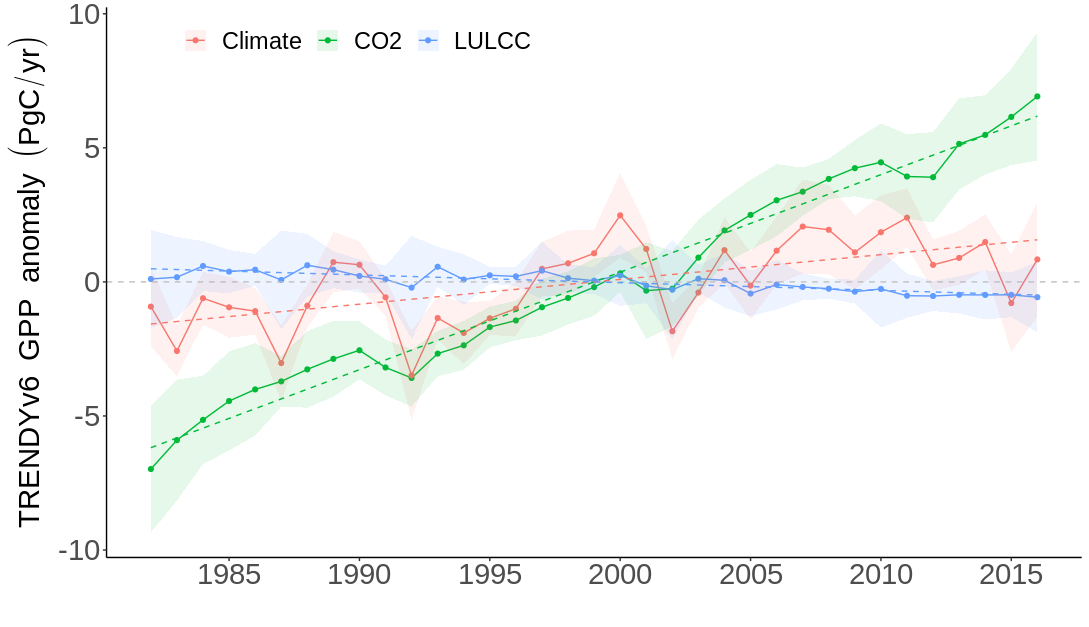


**Figure S1** – Global annual gross primary productivity (GPP) anomalies from TRENDYv6. Annual GPP anomalies (PgC/yr) over the period 1982-2016 estimated from TRENDYv6 models. Simulations S1 (CO2 varies, fixed climate and land cover), S2 (CO2 and climate vary, fixed land cover), and S3 (CO2, climate, and land-use and land cover vary) were used to derive the contribution of each forcing; climate (red), CO2 (green), and LULCC (blue) to global GPP anomalies. “Climate-driven” GPP anomalies are identical to those shown in Figure 1 in the main text. “CO2-driven” GPP anomalies are simply taken from the S1 simulation and “LULCC-driven” GPP anomalies are calculated from the difference between S3 and S2. Linear trends are depicted with a dashed line. Shading represents the 1σ spread among the models.


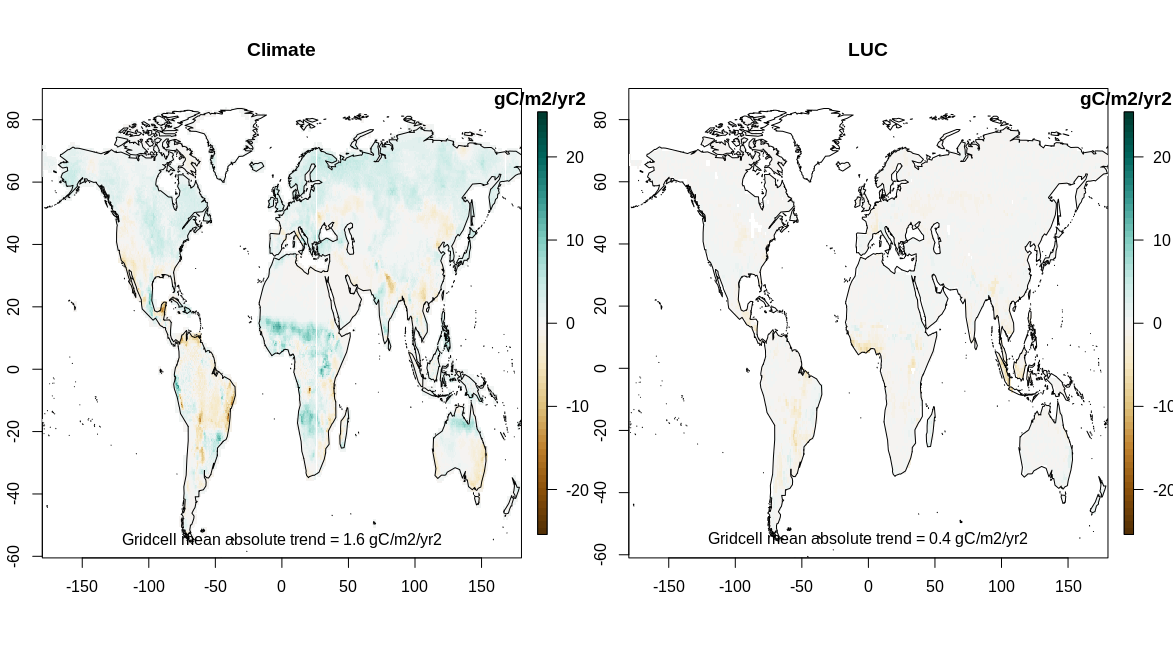


**Figure S2** – Maps depicting the linear trend in annual mean GPP over the period 1982-2016 estimated from the TRENDY DGVMs, due to (left) climate and (right) land-use change (LUC). The gridcell mean absolute trend in GPP due to climate is 4x larger than trends due to LUC. This highlights the relatively minor role LUC has had in overall GPP trends in this time period.


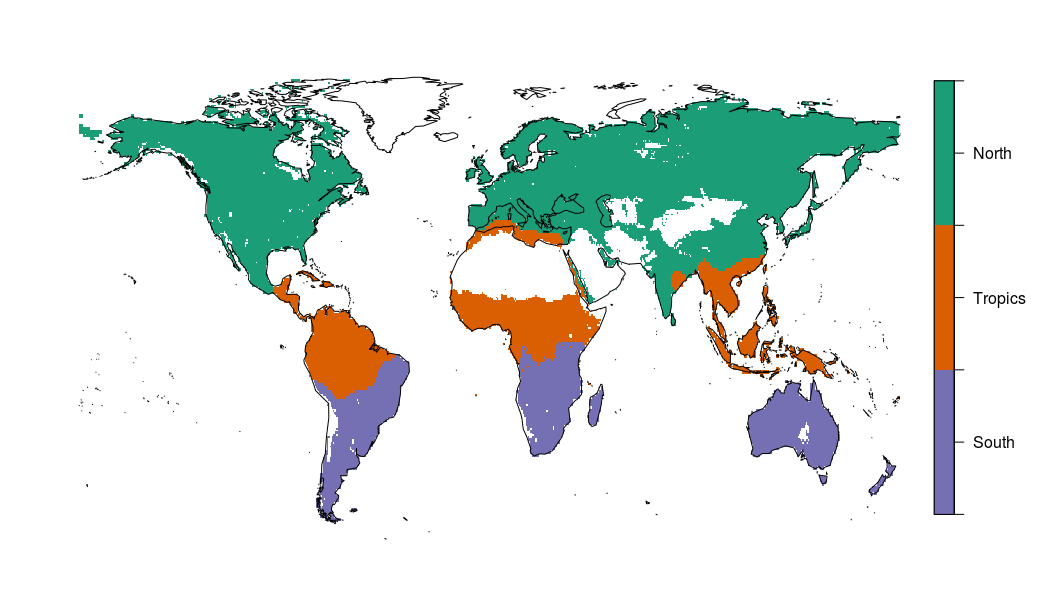


**Figure S3** - Regions used in the study. Regions are broadly defined from the TRANSCOM-3 experiment. North American boreal, North American temperate, Europe, Eurasian Temperate, and Eurasian boreal are grouped as *North.* South American tropical, North Africa, and tropical Asia are grouped as *Tropics.* We further used the MODIS landcover product (MCD12C1) to isolate African tropical forest and included this in the *Tropics* region. Finally, South American temperate, Southern Africa, and Australia are grouped as *South.*


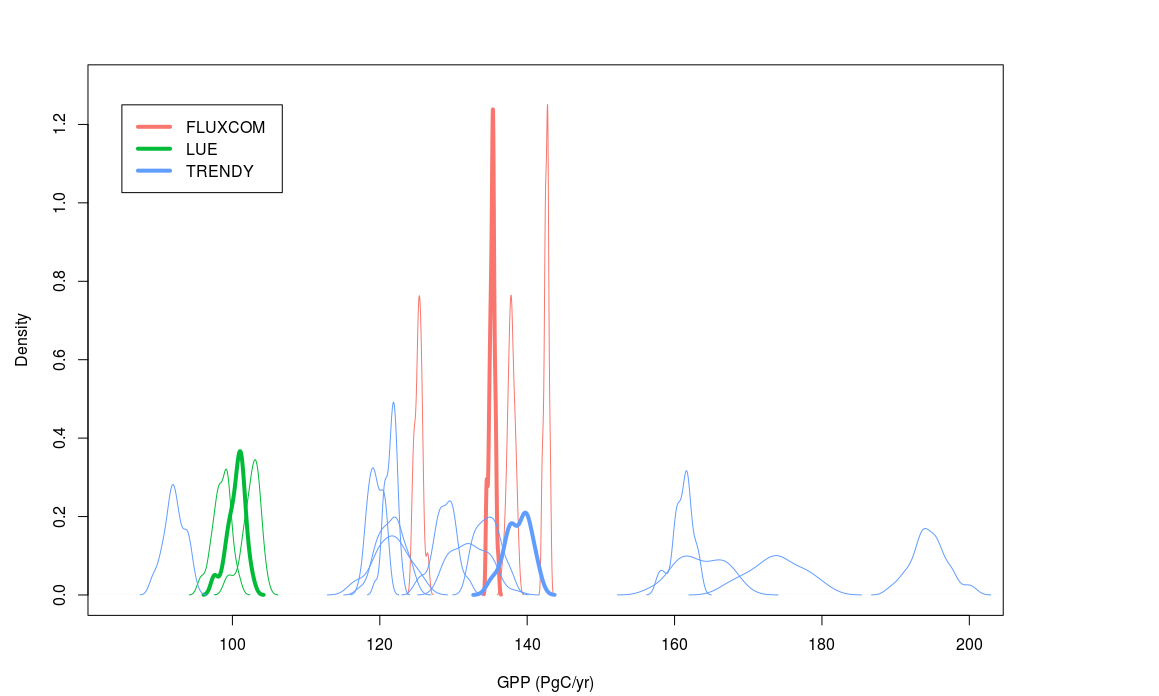


**Figure S4** - Global annual mean GPP distribution (PgC/yr) over 1982-2016 for each ensemble member of the three products. Ensemble means are shown in bold.


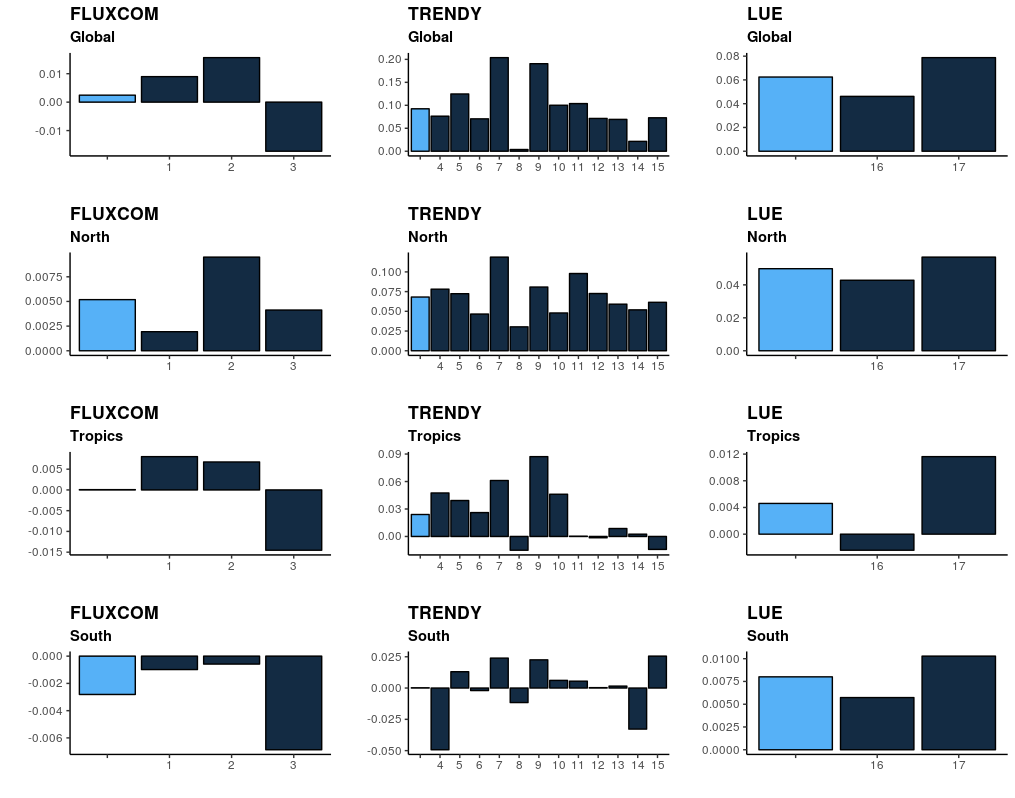


**Figure S5** – Trends in annual GPP (PgC/yr^2^) over 1982-2016 in four regions for each ensemble member from FLUXCOM for three upscaling approaches (Artificial Neural Network – **ANN**, Random Forest – **RF**, and Multivariate Adaptive Regression – **MARS**); (1) ANN, (2) RF, (3) MARS, from TRENDY models; (4) CABLE, (5) CLASS-CTEM, (6) CLM4.5-BGC, (7) DLEM, (8) ISAM, (9) JSBACH, (10) JULES, (11) LPJ-GUESS, (12) ORCHIDEE, (13) ORCHIDEE-MICT, (14) VEGAS, and (15) VISIT, and from LUE model for two parameterizations; (16) Zhao & Running, 2010, and (17) Robinson *et al*., 2018 (references in main manuscript). Ensemble mean trend for each region and product is shown in light blue.


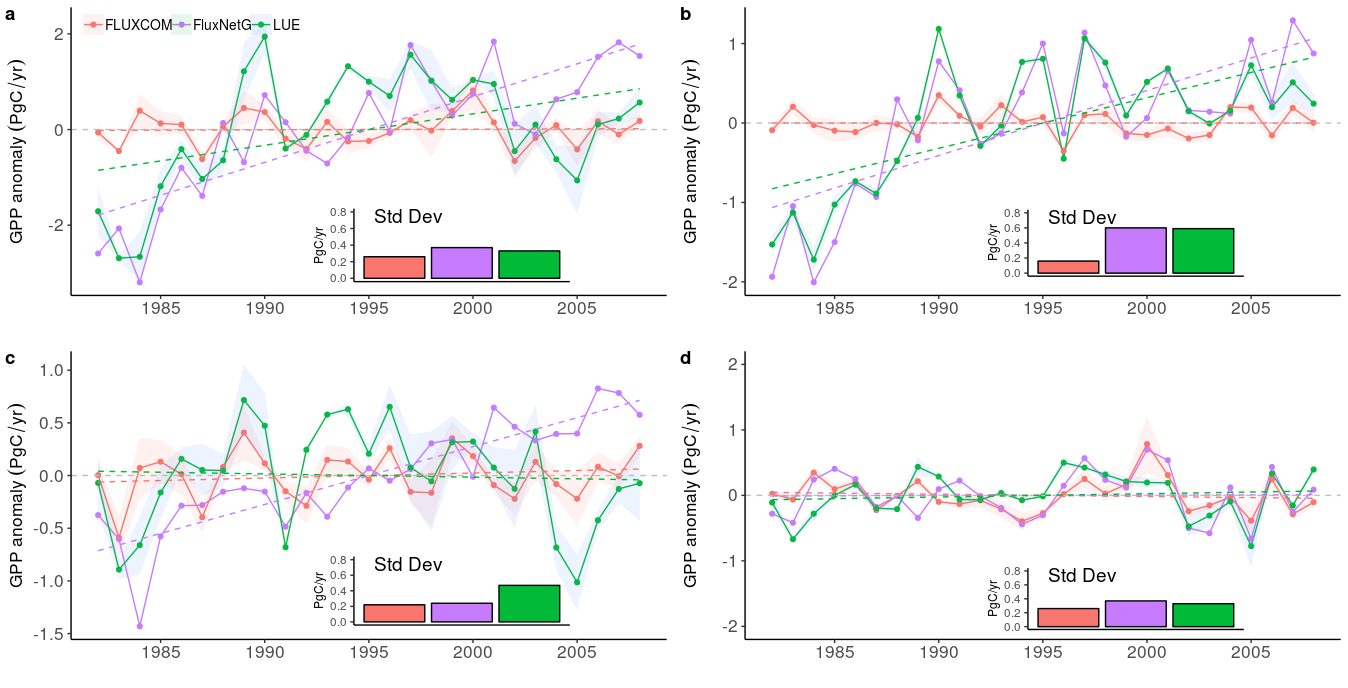


**Figure S6** – Global and regional variations in annual GPP based on three GPP products. Annual GPP anomalies (PgC/yr) over the period 1982-2008 estimated by upscaled flux tower observations, FLUXCOM (red), and FluxNetG (purple), and a satellite-based light use efficiency model (green). Both FLUXCOM and FluxNetG are based on FLUXNET site-level carbon flux observations. However, FLUXCOM is driven with seasonally varying NDVI whereas FluxNetG incorporates interannually varying NDVI in the upscaling procedure. Further, similarly to FluxNetG, LUE is driven with interannually varying satellite data (see Methods). GPP anomalies are shown for a) global, b) northern, c) tropical, and d) southern regions, as defined in Figure S1. Shading represents 1σ spread among each products ensemble members (see Methods). Linear trends are depicted with a dashed line. Bar charts show the inter-annual variability of each product as the 1σ (PgC/yr) of the detrended time-series.


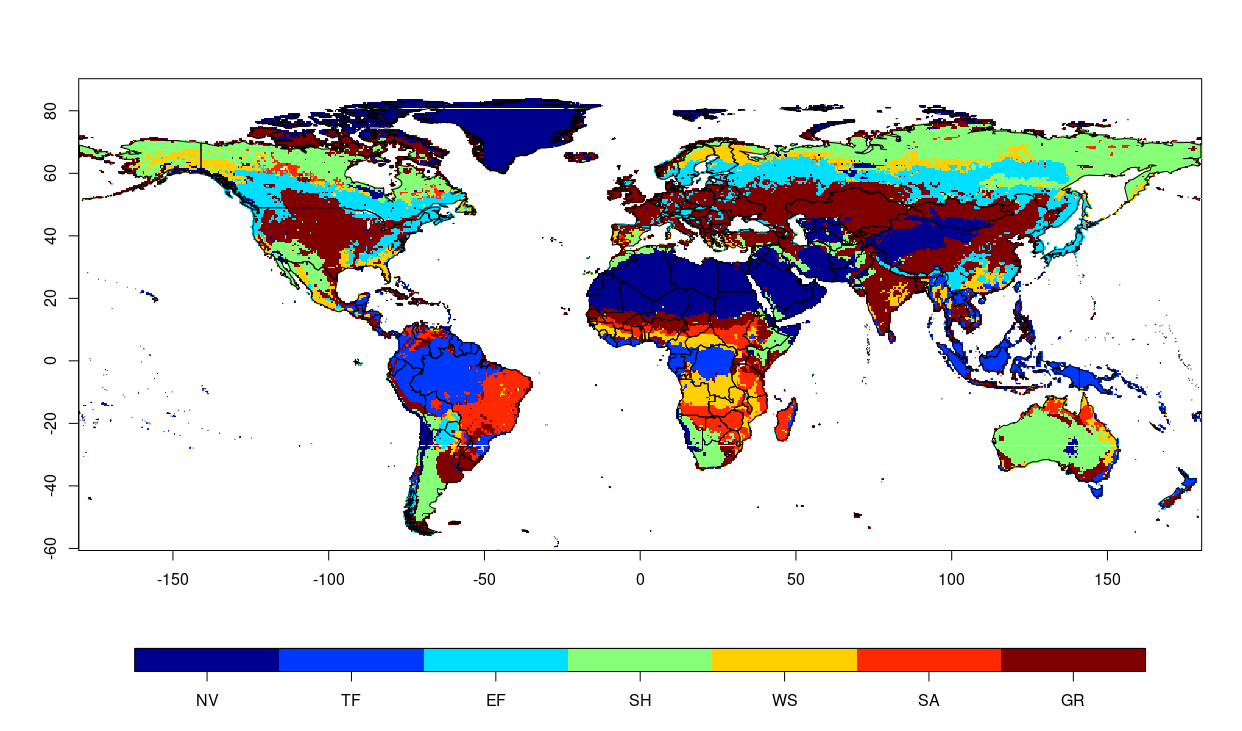


**Figure S7** – Landcover based on the MODIS product (MCD12C1). Merged landcover types include non-vegetated (NV), tropical forest (TF), extratropical forest (EF), shrubs (SH), woody savanna (WS), savanna (SA), and grass/cropland (GR).


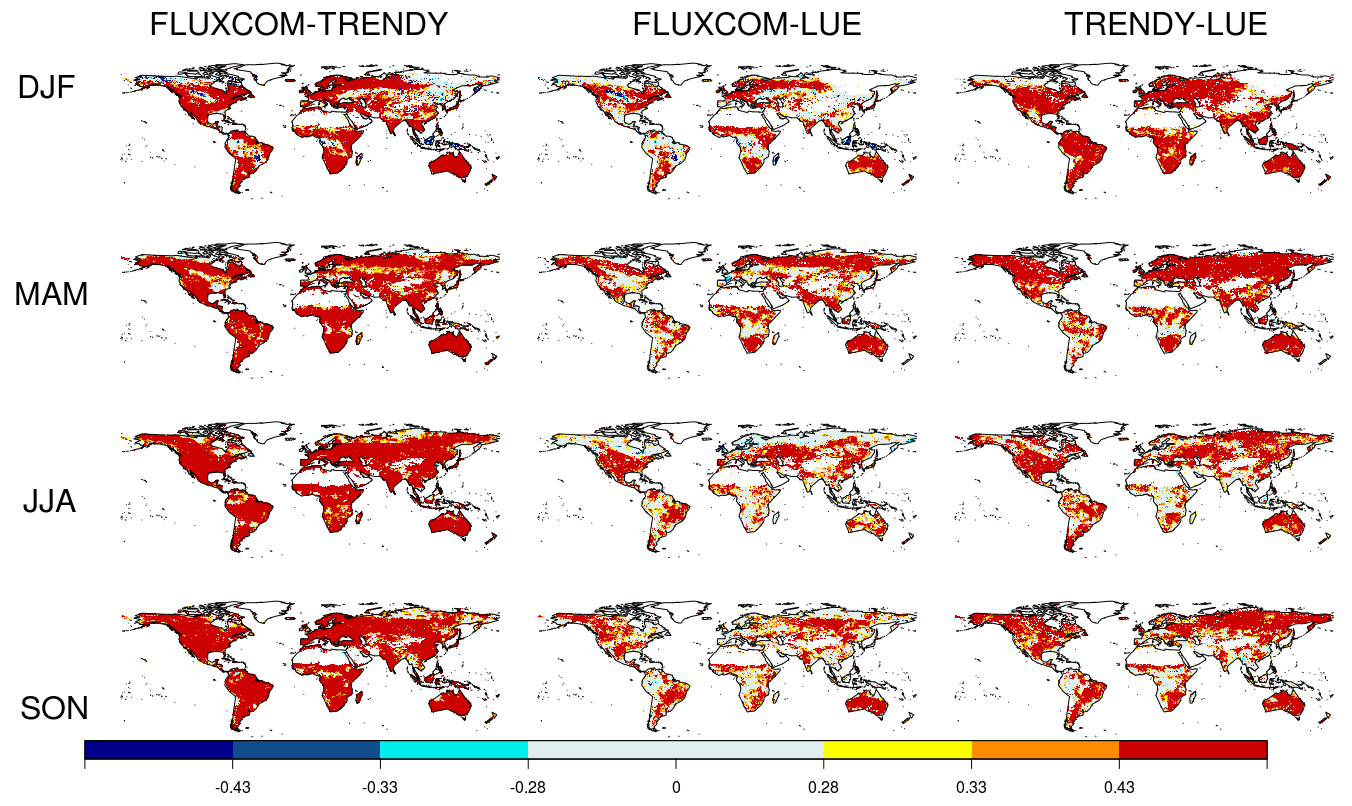


Figure S8 – Correlation between detrended seasonal GPP (1982-2016) for each product. Seasons are defined as December-January-February (DJF), March-April-May (MAM), June-July-August (JJA), and September-October-November (SON). Non-significant correlations (r<0.28, P>0.05) are in grey. Missing data is represented with white. (r=0.28, 0.33, 0.43 corresponds to P=0.1, P=0.05, P=0.01).


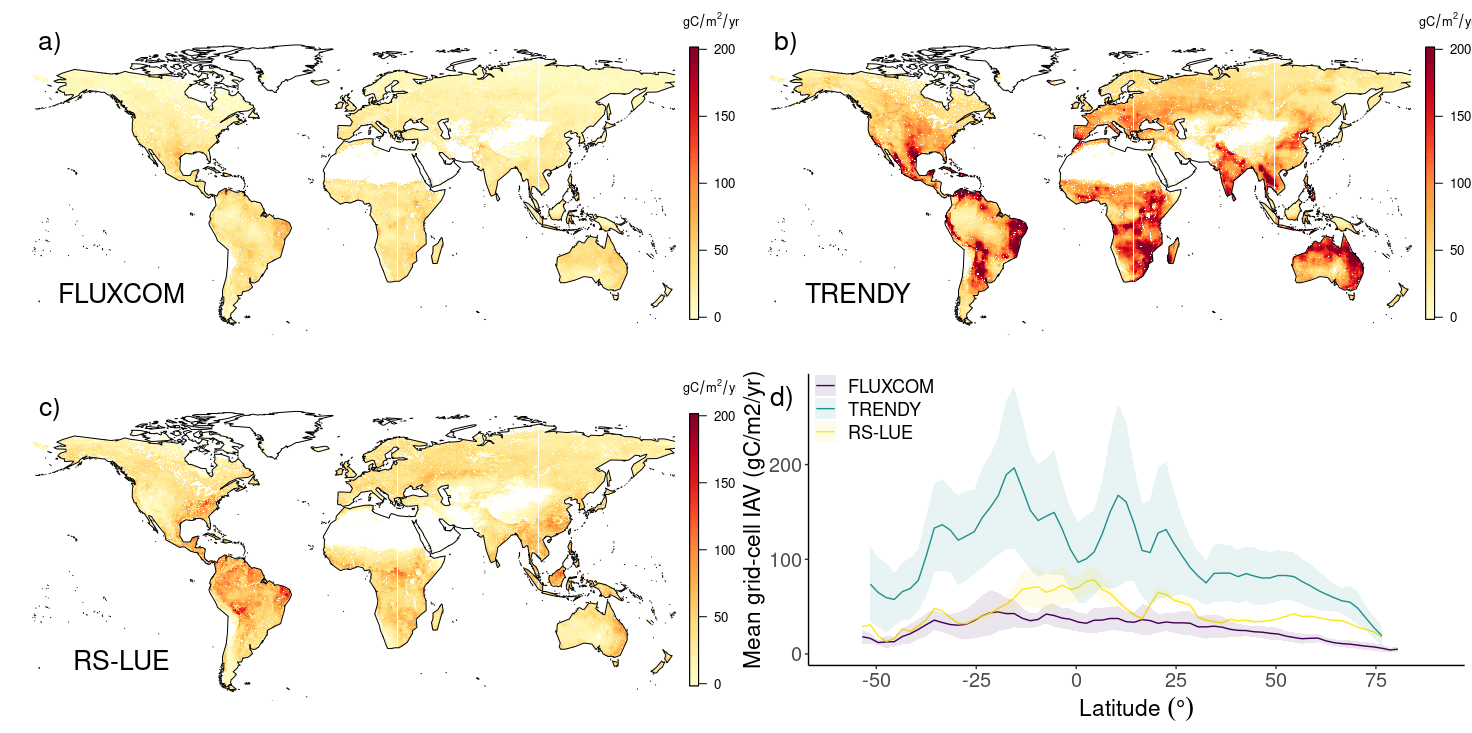


**Figure S9** - Hotspots of interannual variability in GPP (gC/m2/yr). Maps of the magnitude of interannual variability in annual mean GPP over the period 1982-2016 defined for each pixel as the standard deviation in annual mean GPP. Panel d) shows the latitudinal distribution of interannual variability for the three products.


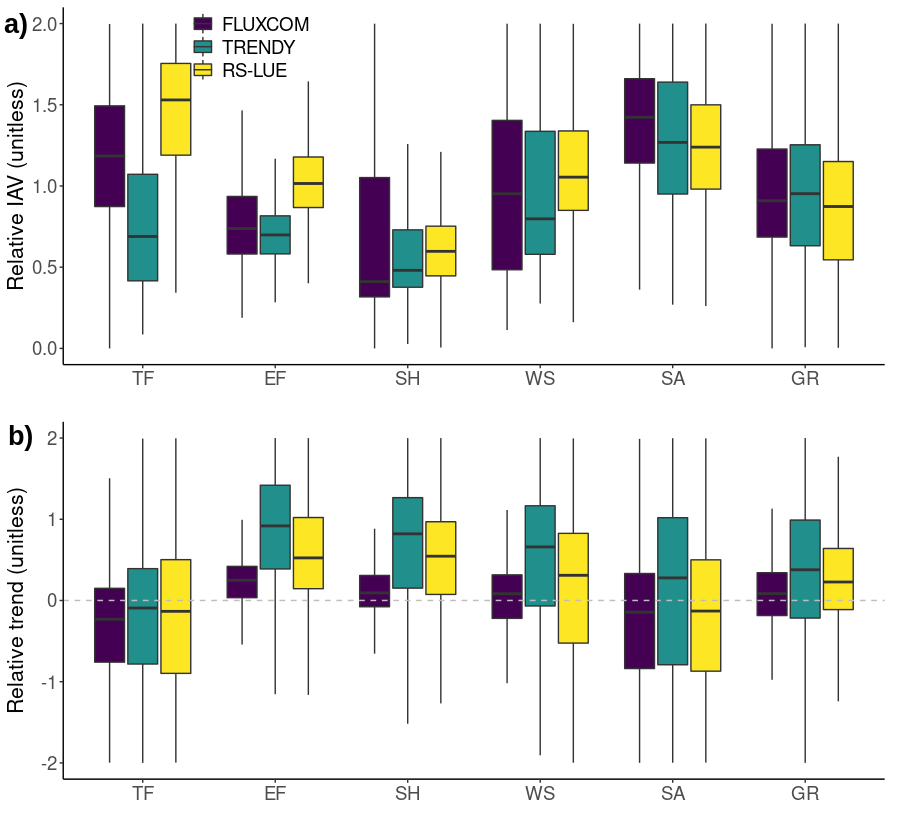
**Figure S10 –** The contribution of different vegetation types to **a)** global interannual variability (IAV) and **b)** global trends in annual mean GPP. IAV and trends are normalised by global mean grid-cell values. Vegetation types are defined in Figure S7.


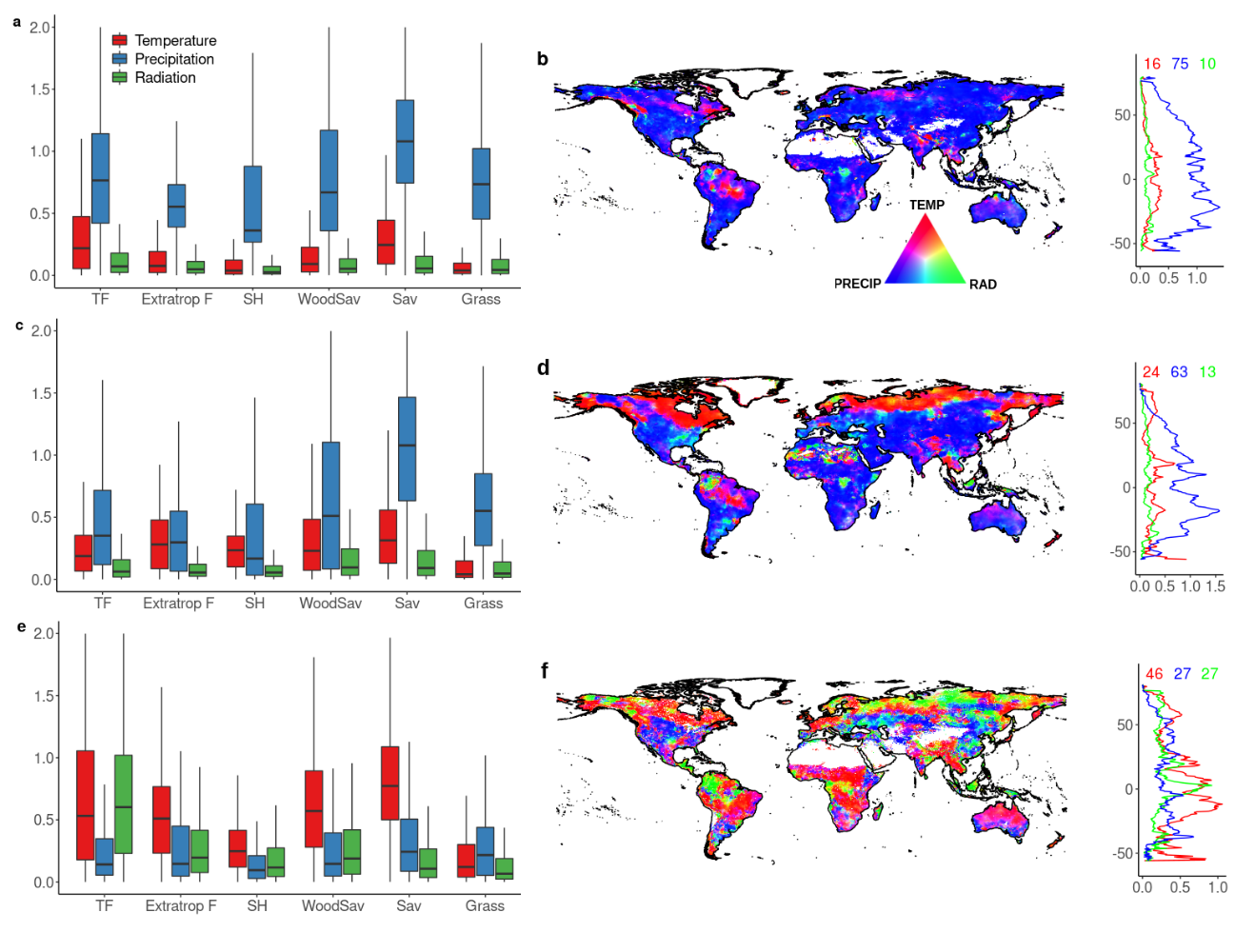
**Figure S11 –** The relative contribution of each climate variable to interannual variability in GPP. Shown are boxplots of grid-cell IAV decomposed into contributions from temperature (red), precipitation (blue), and radiation (green) for different vegetation types for **a)** FLUXCOM, **c)** TRENDYv6, and **e)** RS-LUE. Spatially explicit patterns of the dominant driver (temperature, precipitation, or radiation) of IAV in GPP are also shown. Maps show the relative contribution of temperature, precipitation, and radiation to the explained variance in the multiple linear regression analysis (see Methods) for **b)** FLUXCOM, **d)** TRENDYv6, and **f)** RS-LUE. The contribution of each driver to overall interannual variability (normalised by the standard deviation of globally integrated GPP) is also depicted. Values at top of line plots indicate the contribution (as a percentage) of each climate driver to the variability in global GPP.


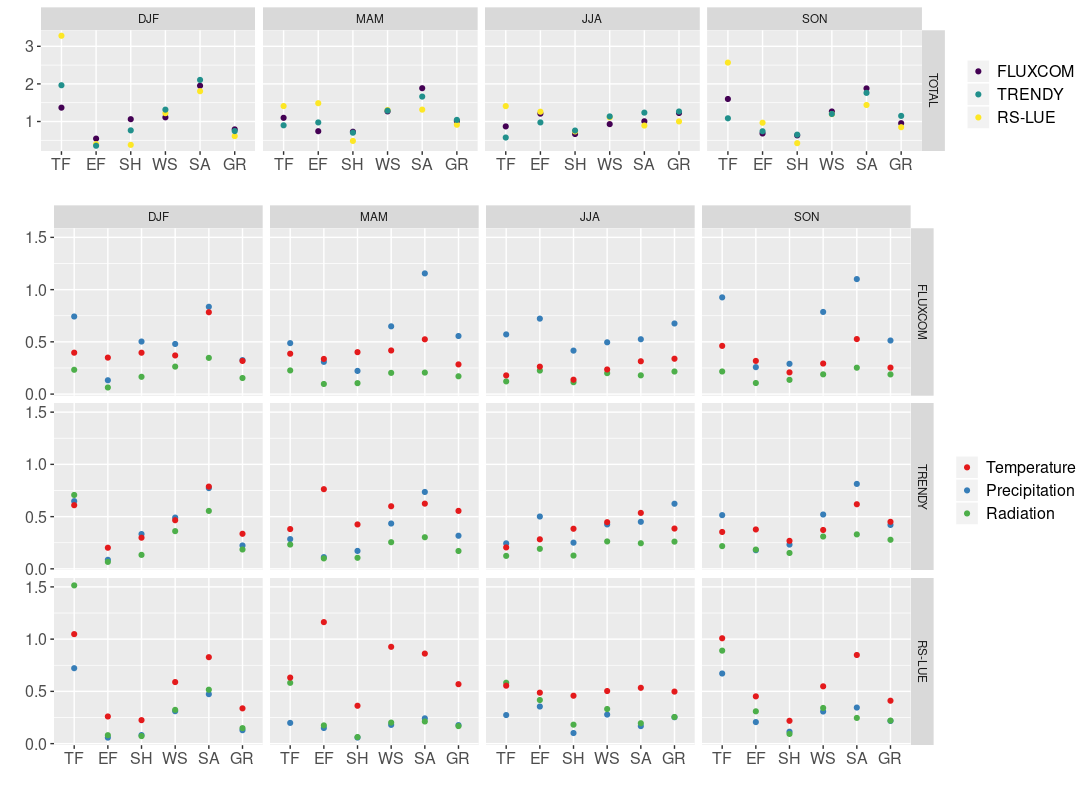
**F****igure S12 –** Interannual variability in seasonal GPP and sensitivity to climate. Top row shows the interannual variability in GPP for each vegetation type (defined in Figure S7), normalised by the global mean variability. The next three rows show the contribution from each climate forcing to the overall variability.


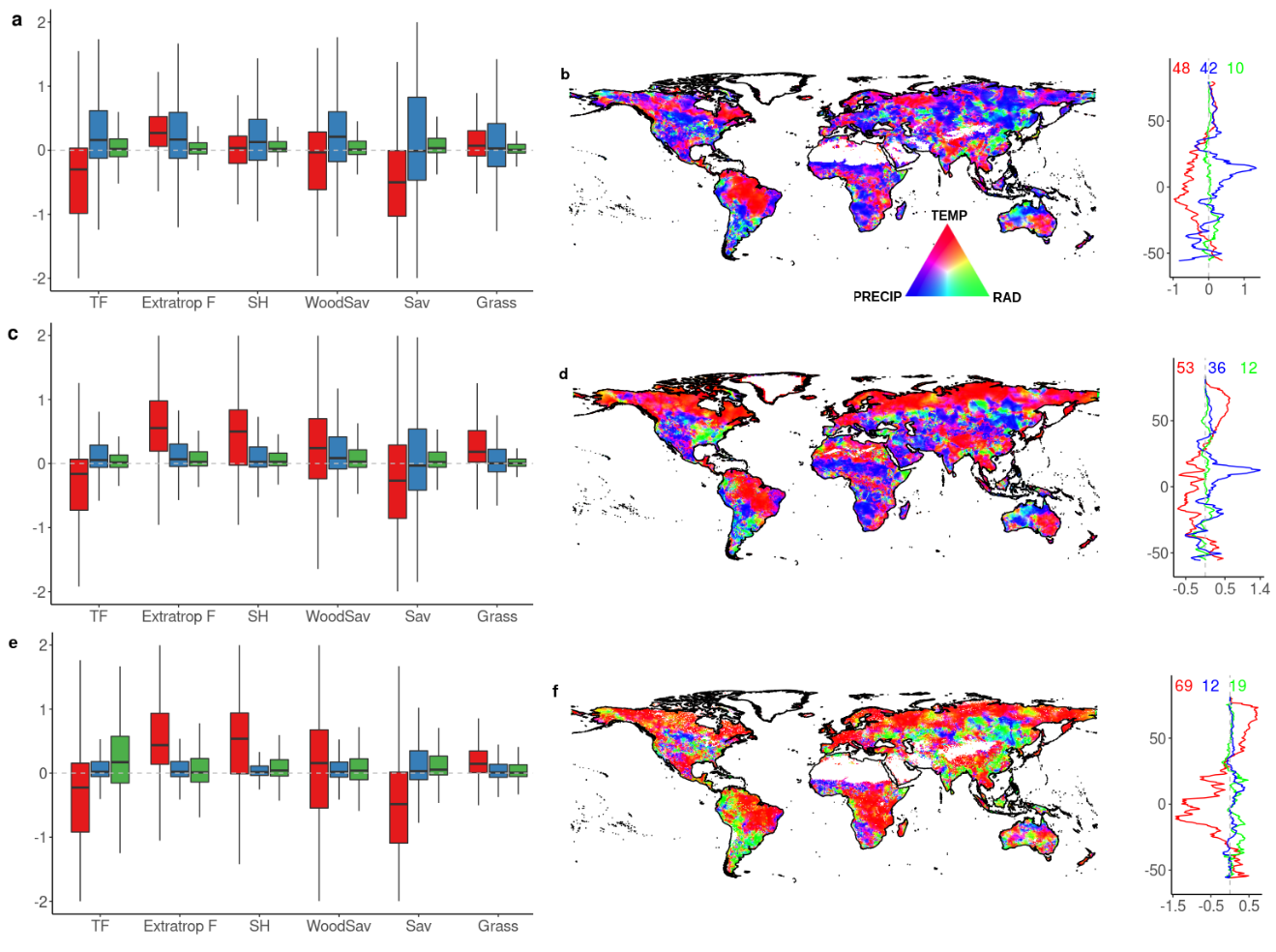


**Figure S13 -** The relative contribution of each climate variable to the long-term trend in GPP over 1982-2016. Shown are boxplots of grid-cell trends decomposed into contributions from temperature (red), precipitation (blue), and radiation (green) for different vegetation types for **a)** FLUXCOM, **c)** TRENDYv6, and **e)** RS-LUE. Spatially explicit patterns of the dominant driver (temperature, precipitation, or radiation) of trends in GPP are also shown. Maps show the relative contribution of temperature, precipitation, and radiation to the explained trend in the multiple linear regression analysis (see Methods) for **b)** FLUXCOM, **d)** TRENDYv6, and **f)** RS-LUE. The contribution of each driver to overall trend (normalised by the standard deviation of globally integrated GPP) is also depicted. Values at top of line plots indicate the contribution (as a percentage) of each climate driver to the trend in global GPP.


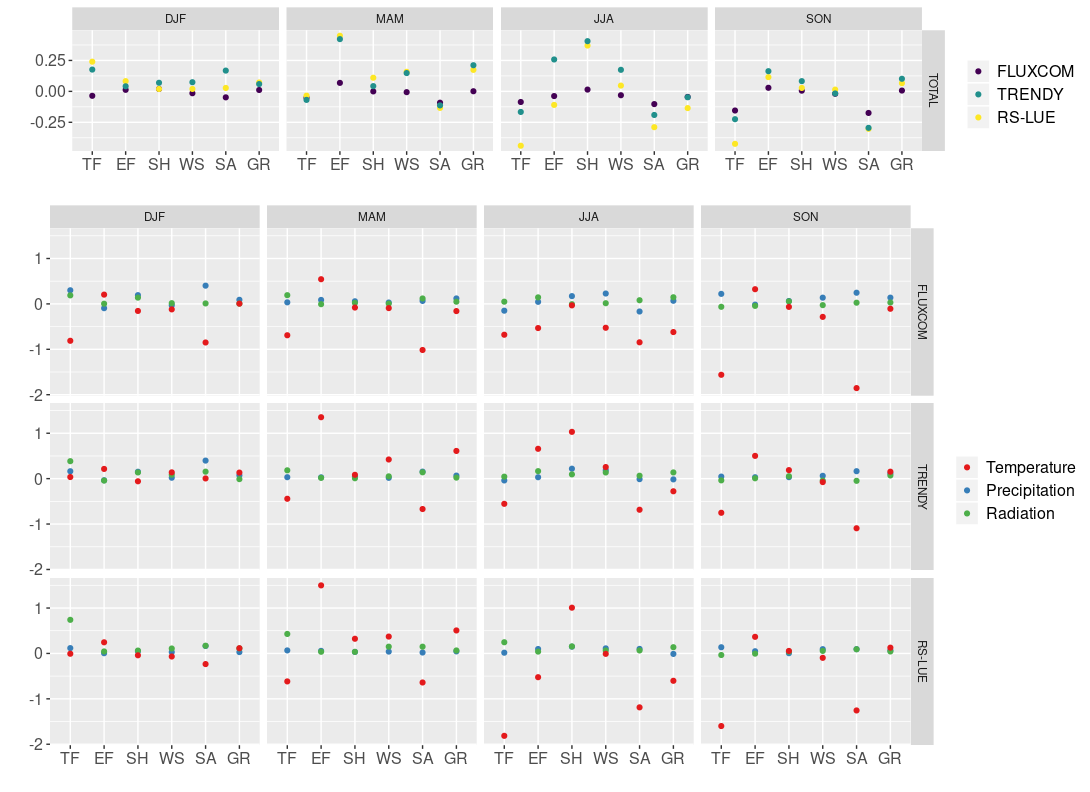
**Figure S14 –** Linear trend in seasonal GPP and sensitivity to climate. Top row shows the trend in GPP for each vegetation type (defined in Figure S7), normalised by the global mean of absolute gridcell trends. The next three rows show the contribution from each climate forcing to the overall trend.


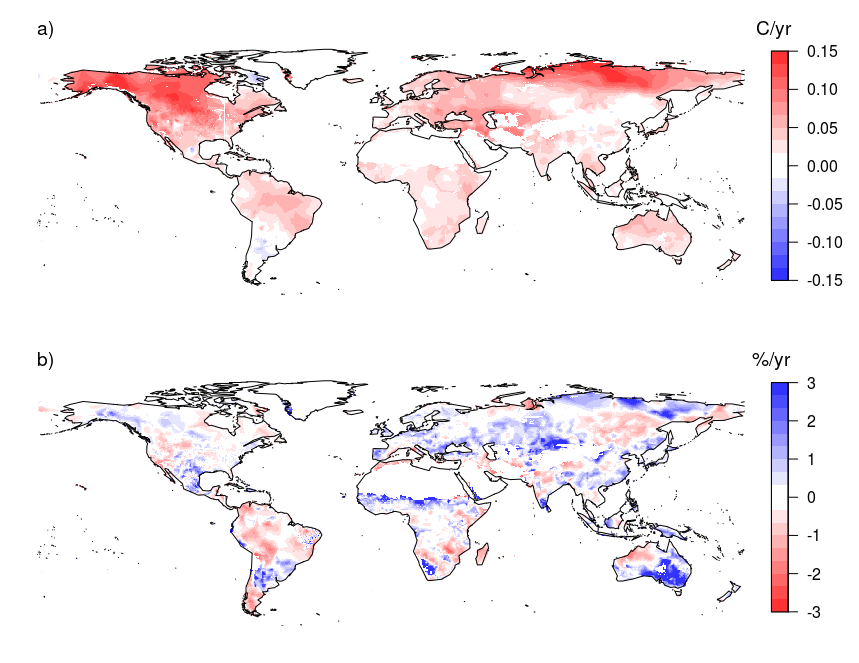


Figure S15 – Linear trends in mean annual temperature (°C/yr) and mean annual precipitation (%/yr) from CRUNCEPv8 reanalysis over the period 1982-2016.
